# DaRA Dataset: Combining Wearable Sensors, Location Tracking, and Process Knowledge for Enhanced Human Activity and Human Context Recognition in Warehousing

**DOI:** 10.3390/s26020739

**Published:** 2026-01-22

**Authors:** Friedrich Niemann, Fernando Moya Rueda, Moh’d Khier Al Kfari, Nilah Ravi Nair, Dustin Schauten, Veronika Kretschmer, Stefan Lüdtke, Alice Kirchheim

**Affiliations:** 1 Chair of Material Handling and Warehousing, TU Dortmund University, LogistikCampus, Joseph-von-Fraunhofer-Str. 2-4, 44227 Dortmund, Germany; nilah.nair@tu-dortmund.de (N.R.N.); alice.kirchheim@tu-dortmund.de (A.K.); 2 Lamarr Institute for Machine Learning and Artificial Intelligence, 44227 Dortmund, Germany; 3 MotionMiners GmbH, 44227 Dortmund, Germany; fernando.moya@motionminers.com (F.M.R.); dustin.schauten@motionminers.com (D.S.); 4 Institute for Visual and Analytic Computing, University of Rostock, 18055 Rostock, Germany; mohd.kfari@uni-rostock.de (M.K.A.K.); stefan.luedtke@uni-rostock.de (S.L.); 5 Fraunhofer Institute for Material Flow and Logistics IML, 44227 Dortmund, Germany; veronika.kretschmer@iml.fraunhofer.de

**Keywords:** dataset, logistics, wearable, inertial measurement unit, Bluetooth, video, third-person view, first-person view, human activity recognition, human context recognition

## Abstract

Understanding human movement in industrial environments requires more than simple step counts—it demands contextual information to interpret activities and enhance workflows. Key factors such as location and process context are essential. However, research on context-sensitive human activity recognition is limited by the lack of publicly available datasets that include both human movement and contextual labels. Our work introduces the DaRA dataset to address this research gap. DaRA comprises over 109 h of video footage, including 32 h from wearable first-person cameras and 77 h from fixed third-person cameras. In a laboratory environment replicating a realistic warehouse, scenarios such as order picking, packaging, unpacking, and storage were captured. The movements of 18 subjects were captured using inertial measurement units, Bluetooth devices for indoor localization, wearable first-person cameras, and fixed third-person cameras. DaRA offers detailed annotations with 12 class categories and 207 class labels covering human movements and contextual information such as process steps and locations. A total of 15 annotators and 8 revisers contributed over 1572 h in annotation and 361 h in revision. High label quality is reflected in Light’s Kappa values ranging from 78.27% to 99.88%. Therefore, DaRA provides a robust, multimodal foundation for human activity and context recognition in industrial settings.

## 1. Introduction

Human activity recognition (HAR) from wearable sensor data is a valuable tool in areas such as sports performance analysis, rehabilitation support or smart homes [1]. Beyond everyday and health-related scenarios, sensor-based HAR is highly relevant in industry. In particular, intralogistics environments present a strong use case, with workers performing tasks such as picking, transporting, packing, unpacking, and storing goods. Here, HAR can serve as a foundation for identifying inefficiencies in workflows, optimizing warehouse layouts, and improving worker ergonomics [2].

The dominant approach to HAR involves mapping sensor signals to activities of interest using machine learning models, such as convolutional neural networks (CNNs) [3,4]. These models rely on well-annotated training datasets. Over the past few years, several datasets targeting manual work in intralogistics have been published, including OpenPack [5,6] and AndyData-lab-onePerson [7,8]. While these datasets have enabled initial progress, their activities are usually restricted to predefined lists of discrete activity classes. In practice, however, many industrial tasks cannot be sufficiently described by a single activity label. Depending on the research or application goal, additional layers of information may be required; e.g., coarse semantic definitions of activities called *attributes* [9] can be used to refer to body postures and sub-activities of both hands, as in the LARa dataset [10], or contextual information, as in the CAARL dataset [11].

The authors of [12] describe activities as entities that take place within a context but can also exist independently of it. Based on this understanding, we distinguish between human movements as the foundation of HAR (e.g., walking, grasping, sitting, bending) and context, which is not strictly required for HAR but can serve as additional information to understand the human movement—human context recognition (HCR). Context includes, among other things, information about the location, time, process, identity, and conditions of subjects and information about the physical environment [13,14]. **Context information** can be categorized into the following:

- **Sensor data**, for example, positional data of subjects and objects;
- **Class labels**, including the location of a subject and its tools, the subject’s process steps, or an order ID;
- **Knowledge**, such as order composition or an ideal process flow.

Existing datasets do not fully capture these aspects. Additionally, these datasets are based on simplified, controlled conditions, limiting their applicability to realistic warehouse operations. This lack of detailed, context-rich data hinders progress toward context-sensitive HAR and HCR in industrial settings.

**Figure A1:**
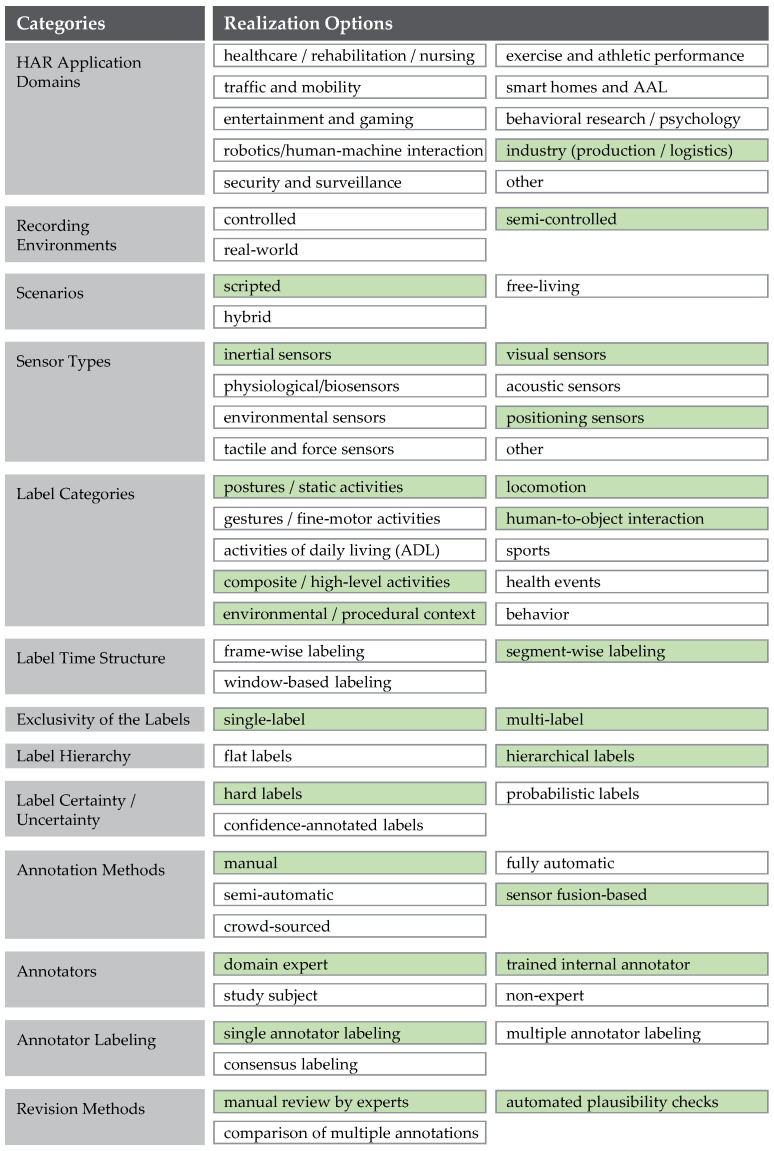
Positioning of the DaRA dataset (green) within the taxonomy of HAR datasets.

To address this gap, we present the **DaRA** (**D**ata Fusion for **a**dvanced **R**esearch in industrial **A**pplications) dataset [15], a novel multimodal dataset for HAR and HCR in intralogistics. DaRA was recorded in a laboratory environment replicating a realistic warehouse, using multiple sensors. It features a distinctive hierarchical annotation scheme with 12 class categories and 207 class labels, covering not only human movements but also contextual information such as location and process stage. This level of detail and quality makes DaRA a unique contribution to the field. An overview of the DaRA dataset is provided in Table 1, and its positioning within the taxonomy of HAR datasets is shown in Figure A1.

## 2. Related Work

The DaRA is a rich dataset for HAR in logistic applications, composed of time-series recordings from Inertial Measurement Units (IMUs), Bluetooth Low Energy (BLE), and videos; it also contains detailed annotations for processes, activities, locations, and movements. This dataset will be relevant for time-series-based HAR, video-based HAR, localization using BLE and process predictions. For justifying and describing DaRA’s characteristics, we dive into HAR methods in logistics environments, HAR and context and HAR datasets.

### 2.1. HAR and Context

HAR can significantly benefit from integrating contextual information, enhancing performance and robustness by leveraging additional data sources that provide insight into the environment or task structure. For example, high-level process states can be used to inform HAR models, improving their ability to distinguish between visually or kinematically similar activities that occur in different contexts [16]. Similarly, location data and information about objects being handled (e.g., picking cart, item, computer) contribute valuable semantic context that refines the outcomes of activity recognition [11]. First-person view approaches for detecting and classifying objects enable more accurate recognition of object-related activities [17].

The authors in [9,18] highlight the importance of semantic attribute annotations for HAR, which support transfer learning and context-aware behavior modeling. Attribute-based activity representations, introduced from computer vision, enable zero-shot learning and class generalization, with approaches using uncertainty sampling and evolutionary algorithms achieving performance comparable to or better than traditional class-based methods [19].

Symbolic HAR methods offer an additional way for incorporating context. They represent human activities and their dependencies using symbolic structures, such as rules, graphs, or ontologies. For instance, some systems model the causal structure of activities with precondition-effect rules. This means certain actions can only happen when specific object states or locations are present [20,21,22]. Contextual information can be integrated into the observation model of these methods, which links sensor input to system states.

### 2.2. HAR Method in Production and Logistics

Production and logistics have human-centered processes, thereby requiring consideration of HAR models that help gain insights into human movements and the ergonomics of individuals. For example, measuring the proportion of different activities during work has been used for optimization tasks such as reducing walking distances or minimizing waiting times for order picking and warehouse processes [9,13,23,24]. Another example is recognizing worker movements, such as bending or carrying heavy items repeatedly, which is beneficial for ergonomic assessment of the worker’s day. Through HAR, such repetitions of these activities can be identified and used to build alert systems that guide workers toward ergonomic practices. Recognition of activities is also relevant for documenting scenarios with repetitive tasks, where a register of activities is to be kept without compromising subjects’ identities [25].

Companies such as MotionMiners (https://www.motionminers.com/, accessed on 18 January 2026) and ProGlove (https://proglove.com/, accessed on 18 January 2026) are already deploying HAR methods in logistics environments. These systems use wearables or handheld devices to capture worker movements and provide task-specific assistance.

### 2.3. HAR Datasets

The majority of publicly available HAR datasets are focused on three application domains: healthcare/rehabilitation/nursing, exercise and athletic performance, and smart homes and Ambient Assisted Living (AAL) [26]. These domains primarily encompass the recognition of the following:

- Activities of Daily Living (ADL) [27] like cooking, eating and drinking, sleep behavior, and step counting (e.g., *Daily Log* [28,29], *ILMHAR* [30,31]), *SLAM HAR* [32,33]);
- Locomotion (e.g., *RealWorld* [34,35], *UMAFall* [36,37], *HuGaDB* [38,39]);
- Gestures (e.g., *HCI gestures* [40,41], *Hand Gesture* [42,43], *LaRED* [44], *HaGRID* [45,46]);
- Dancing (e.g., *3DLife/Huawei ACM MM Grand Challenge 2011* [47,48], *HDM12 Dance* [49], *Martial Arts, Dancing and Sports (MADS) Dataset* [50,51]);
- The analysis of sports activities (e.g., *BodyAttack Fitness* [40,41], *UMONS-TAICHI* [52,53], *UCF Sports* [54,55], *Hang-Time* [56,57]);
- Fall detection, particularly in individuals with physical impairments (e.g., *UMAFall* [36,37], *Teruel-Fall (tFall)* [58,59], *SisFall* [60,61], *Fall-UP* [62,63]).

In contrast, other application domains of HAR, such as traffic and mobility, entertainment and gaming, behavioral research and psychology, robotics and human–machine interaction, as well as security and surveillance, are comparatively underrepresented in the freely available datasets. The industry domain, comprising production and logistics, has become increasingly relevant since 2017, leading to a growing number of available datasets in this field (see Table 2).

Table 2 presents a brief survey of datasets for industrial settings since 2008. References for the datasets and dataset website links are provided where available. The year of publication, the public availability status, the dataset size, the number and types of sensors used, and the number of subjects who participated in the recording process are noted. Depending on how each dataset creator described it, the dataset size is presented as hours of recordings, as the memory utilized by the dataset, or, in the case of IHADv, as the number of images. Sensor types can be visual, such as MoCap, RGB, and RGB-D sensors, or non-visual, such as inertial, biosensors, and tactile sensors. The number of sensors refers to the devices placed on the human or in the environment. In MoCap, the number of sensors refers to the number of cameras used during recording. Four categories of recording environments were identified, namely, real-world, semi-controlled, controlled, and virtual. Controlled environments refer to laboratory settings, while semi-controlled environments can be a sensor setup within a real-world scene. In the unique case of InHARD-DT [71], subjects’ movements were recorded during their interactions with the virtual reality scene. This category provides insight into the fluidity and realness of movement performed by the individuals. Next, the label category, number, and annotation type are addressed. Label category is based on whether the labels focus on posture, human–object interactions, human–robot interactions, ADLs, or sports. In the *label number and type* column, the availability of coarse labels is noted where available. For instance, one can provide broad activity labels, such as walking, running, and holding a box, or finer labels, such as using the left hand, the right hand, or a small item in hand. The final column, *Annotation*, refers to who or how the activities were labeled in the dataset. It could be domain expert annotation, manual annotation with required subjects, auto-labeling by the subject performing the activity, or no annotation effort, as the activities are conducted in a protocol-defined manner.

The table shows that there has been an increase in human–object and human–robot interactions, whereas the initial datasets focus on human posture in industrial contexts [13]. Each dataset is unique in its sensor selection, number of subjects, recorded human movement information, and activity class labels. Only four datasets have more than 40 subjects. Similarly, only four datasets are based on real-world environments. The most interesting part of the table is the *Labels* categories. It can be noted that most datasets focus solely on activity classes. Very few works have focused on presenting coarse actions or semantic information. Even fewer have included contextual information.

Though these datasets broadly cover industrial movements, they do not include all possible movements within the industrial context. For instance, the movements included in packaging differ from those in order picking, and the movements in car assembly differ from those in Activities of Daily Living. From Table 2, we see that a few datasets, such as IKEA ASM [93] and Skoda Mini Checkpoint [101], focus on assembly movements, while few others, such as Physical Human–Robot Contact Detection [81] and COVERED [77], focus on human–robot collaboration scenarios that are of interest in the future of industrial settings. Datasets such as OpenPack [5], LARa [10], and CAARL [79] specifically focus on logistics scenarios such as packaging and order picking. The movements included in these datasets are closest to those presented in DaRA. While LARa and CAARL were recorded with a focus on MoCap and IMU sensors, OpenPack includes IMU, blood volume pulse, electrodermal activity, LiDAR, and depth image sensors. Further, OpenPack focuses solely on the packaging scenario and doesn’t include order picking, whereas LARa and CAARL include both, with order picking given priority. While OpenPack, LARa, and CAARL have coarse labels, the label types differ across them. LARa and CAARL have an action-class and attribute-label structure. This means that the action class standing has an attribute representation that denotes whether the standing action is still or with small-step motions, whether the item is in the left, right, or both hands, and the size of the item. However, in OpenPack, the annotation is used on the operation performed and its sub-action classes. Thus, the close box operation has the subclasses *bend flap* and *attach tape*. However, the subactions do not span the entire operation. Actions that are in between these subclasses are not always labeled.

### 2.4. Research Gaps

Although the number of available datasets has been steadily increasing, there remains a significant shortage of datasets that reflect realistic recording and working conditions, contain rich metadata, provide comprehensive contextual sensor data, and include annotated contextual class labels. Without contextual information, the interpretation of recognized activities, the description of workflows, the identification of errors, and the derivation of optimization measures are severely limited or even impossible, which undermines the primary objective of HAR and HCR in the industrial domain.

Inconsistencies in labeling, inadequate dataset documentation, and restricted data accessibility further undermine comparability, generalization, and reproducibility, ultimately limiting practical applicability. The movements or activity annotation labels in industrial datasets available are specific to the task focused upon; for example, in Assembly 101 [76] (see Table 2), the coarse labels were *attach track* or *attach cabin*, while the fine labels were *picked up the chassis* or *screw track with a hand*. These labels are difficult to transfer to different scenarios, even when the action performed is similar. Consequently, more datasets addressing human motion in various industrial settings are of interest. In [80,97,102], the movements were made for the respective annotation labels. Therefore, motion continuity could be missing unless the dataset is focused on recording continuous activities in the scenario. Although repeating the same activity is intended to simplify the annotation process and ensure balanced activity class recordings, this practice is detrimental to motion variability.

Consequently, DaRA has continuous movements, where the subjects performing the activities are oblivious to the annotation labels, and, thereby, annotators of the DaRA dataset had the excruciating task of identifying transitions from one activity to another while labeling. This dataset further facilitates the study of learning jitter in annotation labels and how to address transitions in a movement. With the detailed annotation label and contextual data available, it is possible to extend the annotation label into detailed textual data, which can later be used to annotate movements with similar characteristics.

## 3. DaRA Dataset

This industrial dataset, focused on logistics activities of order picking and packaging in a semi-controlled laboratory environment, was created following the checklist in [103]. The dataset description follows the approach proposed by [104]. To ensure compliance with the FAIR principles [105], the data was made easily **f**indable and **a**ccessible on Zenodo, **i**nteroperable, and **r**eusable with the availability of metadata in this paper and in the documentation on Zenodo.

**Figure 1:**
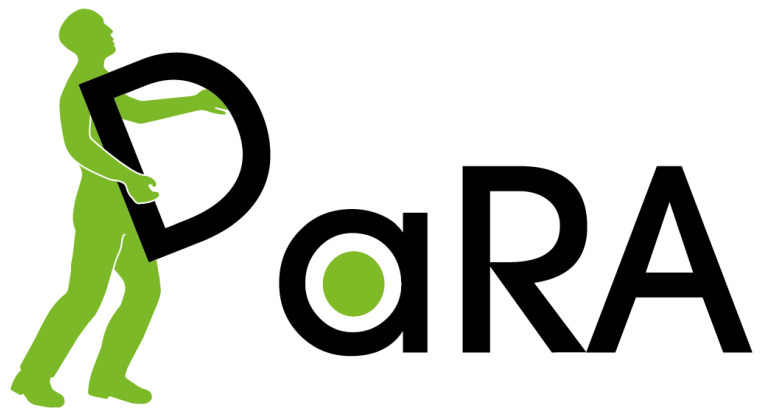
Logo of the DaRA dataset. At the core of the dataset and the logo is the human at work, manipulating and transporting objects in warehouses.

This section presents the results of the dataset creation process and the specifications of the dataset **DaRA** [15]; see Figure 1. First, the experimental setup is described, including the laboratory environment, scenarios, and sensors used. Next, the selection of subjects is explained, followed by a detailed description of the data collection process. Subsequently, the 12 different class categories are introduced and assigned to their respective class labels. This is followed by an explanation of the annotation and revision process.

The dataset quality is evaluated based on annotation consistency, device data loss rate, and a use case, i.e., solving HAR for DaRA’s main and sub-activities. Finally, we provide guidance on how to use the dataset effectively. We provide a Python script (version 1) [106] that allows users to customize the annotation results to extract precise information required for their specific use case.

### 3.1. Experimental Setup

The following sections describe the laboratory where the recordings took place, the eight logistics scenarios, and the three types of sensors used.

#### 3.1.1. Introduction to the Laboratory Picking Lab

**Figure 2:**
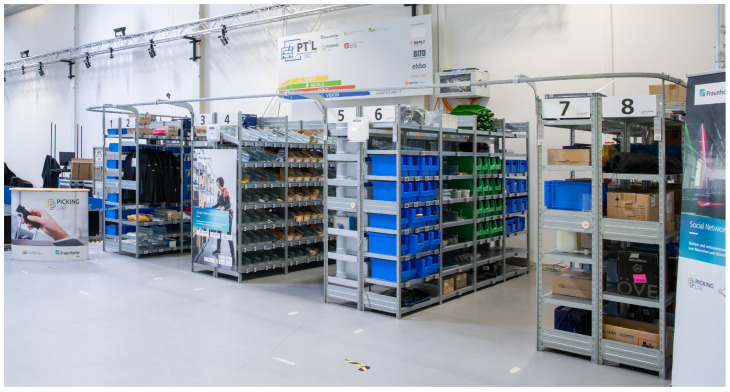
Picking Lab at the Fraunhofer Institute for Material Flow and Logistics (IML) in Dortmund, Germany. The photo shows eight numbered industrial rack complexes. The rack complex 1 stores small items in open-fronted storage bins. Complexes 2 and 3 hold hanging goods and loose items without bins. Complex 4 features flow channels for unboxed items and those in cardboard boxes. Complex 5 contains medium-sized flat goods, while complex 6 mainly stores medium-sized to bulky flat goods in green and blue open-fronted storage bins. Bulky and heavy items are located primarily in complexes 7 and 8.

The experimental setup was established in the Picking Lab at the Fraunhofer Institute for Material Flow and Logistics IML (https://www.iml.fraunhofer.de/en/fields_of_activity/material-flow-systems/intralogistics_and_it_planning/services/Picking_Lab.html, accessed on 18 January 2026). The Picking Lab is a research infrastructure designed for application-oriented logistics research [107]. It focuses on key questions such as process optimization, logistical information technology (IT), human–technology interaction, and ergonomics [108]. The lab replicates a small-scale order picking warehouse (see Figure 2) and is specifically designed to evaluate technologies and processes in the context of conventional order picking systems based on the person-to-goods principle. This environment allows the investigation of both technological and procedural aspects of order fulfilment. It can be considered semi-controlled because it presents a realistic warehouse that replicates essential technical and logistical characteristics of an authentic warehouse within a controlled laboratory environment.

**Figure 3:**
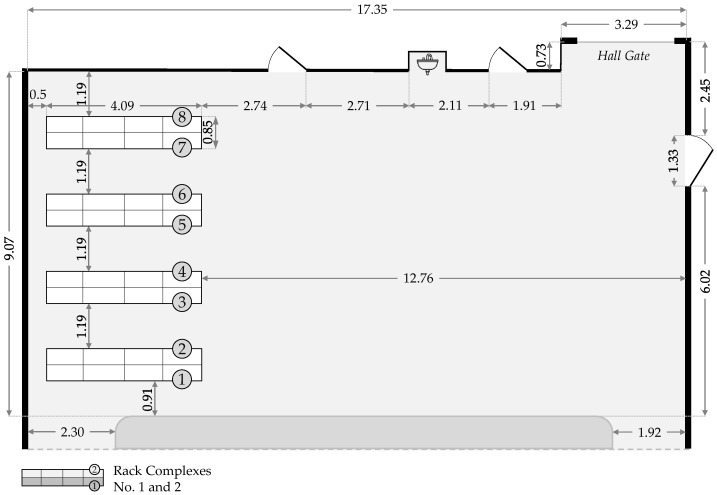
Floor plan of the Picking Lab, showing the eight rack complexes and the open area in front of the racks used for workflow simulation. All measurements are given in meters.

The standardized environment consists of eight rack complexes across five aisles, complemented by an open area in front of the rack storage system (see Figure 3). This configuration enables the implementation of realistic scenarios for typical intralogistics applications, including e-commerce, small-parts picking, and handling bulky or hanging goods.

A wide range of items is available for handling:

- Small items (from 0.4 g), such as screws, locknuts, washers, or bits;
- Medium items (approximately 50 to 800 g), such as softshell jackets, ties, gloves, hoodies, bags, shirts, or notebooks;
- Large items (up to 5149 g), such as palm soil, axes, and hacksaws.

The item master data, including dimensions, weight, designation, storage location, item photographs, and customer orders, are documented and accessible on Zenodo (see *Documentation.pdf* file).

The items are stored in compartments, such as small load carriers, open-fronted storage bins, without bins, cartons, hanging rails with clothes hangers, or flow channels, according to their characteristics. Electronic rack labels are used for identification. The Picking Lab is equipped with a cloud-based warehouse management system (WMS) that interfaces with the IT systems of the picking technologies.

#### 3.1.2. Logistics Scenarios

**Laboratory Layout and Scenario Integration**

**Figure 4:**
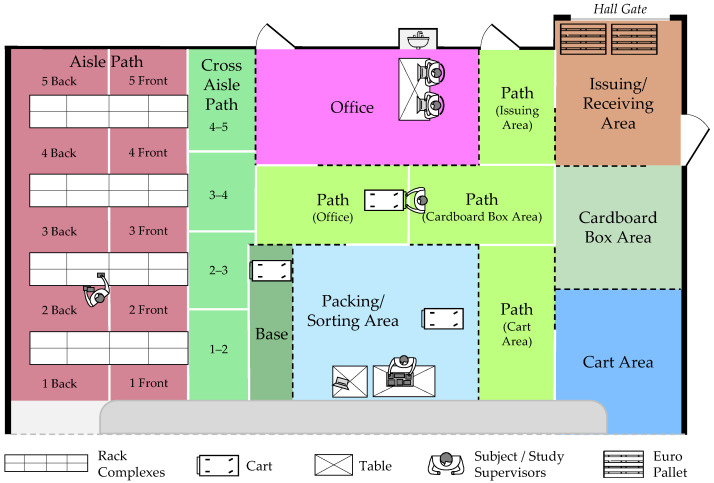
Floor plan of the Picking Lab. The entire setup is color-coded into nine main areas. The colors correspond to the annotation tool SARA annotation tool’s coding scheme [88,109]. The dashed black lines represent physical boundaries formed by barrier stands with belt straps, while the solid white lines indicate conceptual boundaries marked by tape on the floor.

The open area of the Picking Lab was divided into distinct work zones. Including the *Aisle Path* within the rack storage system, the lab comprises nine main areas (see Figure 4). Additionally, the *Aisle Path*, *Cross Aisle Path*, and *Path* were subdivided into further zones to cover detailed process steps.

**Realistic Material Flow Integration**

**Figure 5:**
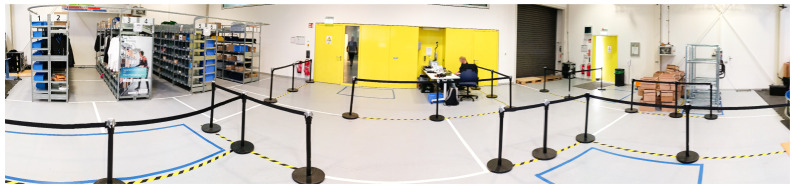
Panorama view of the warehouse setup. The photo was taken in the *Packing/Sorting Area* (light blue area in Figure 4) behind the packing table, with a view towards the *Office* (pink area in Figure 4). Within the office, the study supervisor is seated at a desk, from which subjects receive their assignments. Behind the supervisor, to the right from the photo’s perspective, is the *Issuing/Receiving Area* by the black hall gate. The boxes in front of it indicate the *Cardboard Box Area*. The *Cart Area* is situated further ahead, where three picking carts are located. These areas are connected by a 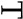 shaped path. On the left side of the photograph, the *Base*, *Cross Aisle Paths*, and the Picking Lab with its eight rack complexes and five *Aisle Paths* are visible.

In contrast to the isolated processes typically represented in state-of-the-art datasets, this study implemented a holistic, realistic warehouse-specific material flow. During each recording session, three subjects simultaneously traversed the entire material flow, as illustrated in Figure 4. Supervisors acted as warehouse managers, located primarily in the *Office*, where they assigned orders and managed information technology, accepted returns upon order completion, and assisted subjects when needed. The experimental setup is depicted in Figure 5.

**Overview of Scenarios**

A total of eight scenarios were implemented. They differ in terms of the high-level process (retrieval vs. storage), the IT used, customer orders, picking strategies, and intentional errors in the picking lists. Scenarios 1–3 and 7 focused on retrieval, while scenarios 4–6 and 8 focused on storage. As in a real warehouse, the process steps in the scenarios were predefined, but the movements required to perform them were not prescribed to the subjects, allowing for realistic motion.

 

**Retrieval (Scenarios 1–3)**

Retrieval scenarios started with order preparation. Subjects received their picking orders and assigned information technologies in the *Office* area. They proceeded via the *Path* to the *Cart Area* to select a picking cart, then collected empty cardboard boxes in the *Cardboard Box Area*.

Picking began with transporting the cart to the *Base*, where it remained during the picking process. Items were put into cardboard boxes on the cart, reflecting a Pick & Pack approach where items are picked directly into shipping-ready boxes.

**Figure 6:**
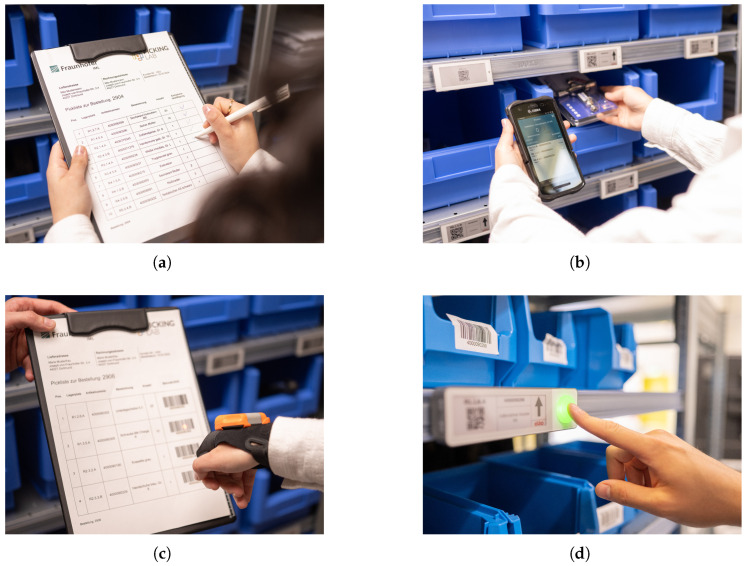
Information technology for guiding the picker: (**a**) picking list with pen, (**b**) portable data terminal, (**c**) picking list with glove scanner, (**d**) Pick-by-Light signal.

The picking strategy followed a single-order picking principle: each picking task corresponded exactly to one customer order. This straightforward, order-oriented approach did not require further sorting or consolidation. Information relevant to order fulfillment, such as item identifiers, required quantities, and storage locations, was provided to the picker through different forms of guidance and confirmation media. In this paper, these means of information provision are collectively referred to as *information technologies* (Figure 6):

1. Scenario 1: Paper list with pen.
2. Scenario 2: Portable Data Terminal (PDT).
3. Scenario 3: Paper list with glove scanner.

Subjects moved between their carts in the *Base* and the respective item positions within the *Aisle Path*, following a return-aisle strategy, where aisles were entered and exited at the same end repeatedly. For scenarios 1–3, subjects were instructed to enter each aisle separately for each position of the order, without processing multiple positions simultaneously.

In scenarios 2 and 3, a *pick-by-light* system guided subjects, using optical signals on rack complexes and storage compartments to the correct items. While the light indicated the picking location, the number of items to be picked was displayed on either the list or the PDT.

Once all items for an order were picked and placed into the boxes on the cart, the cart was transported to the *Packaging Area*, where packaging materials (e.g., bubble wrap, shipping labels, delivery notes, box cutters, and tape) were provided. After packaging, orders were finalized by transporting the boxes to the *Issuing/Receiving Area*, where they were placed on pallets. Finally, subjects returned their IT (picking list, pen, portable data terminal, glove scanner) at the *Office*.

Each subject repeated this retrieval process three times, handling different customer orders (order IDs: *2904*, *2905*, *2906*) and employing different information technologies in each iteration.

 

**Intentional Errors in Picking Lists**

To reflect realistic warehouse processes, the scenarios intentionally included disruptive elements. Planned errors included incorrect storage locations on picking lists (scenario 1), quantity discrepancies (scenario 3), inappropriate box sizes, waiting times due to limited packaging stations, and missing materials, such as plastic bags, which had to be retrieved from the *Office*. Additionally, unplanned errors occurred, such as device handling mistakes or quantity and type errors during picking or storage.

 

**Storage (Scenarios 4–6)**

Following the retrieval runs, storage processes were conducted in scenarios 4–6. These began with order acceptance in the *Office* and goods receipt in the *Issuing/Receiving Area*. At this stage, previously completed retrieval orders (processed three times each) were placed on pallets.

Each subject processed one storage order three times. After transportation to the *Packaging/Sorting Area*, boxes were unpacked, and items were sorted for storage. Storage involved placing items into the rack storage system, guided exclusively by paper lists with pens.

Upon completing storage, subjects finalized their orders by returning empty boxes to the *Cardboard Box Area*, their lists and pens to the *Office*, and the carts to the *Cart Area*.

 

**Multi-Order Picking (Scenarios 7–8)**

Scenarios 7 (retrieval) and 8 (storage) followed the same structure as the previous scenarios but introduced multi-order picking. Two customer orders (*2904* and *2905*) were processed in parallel within a single picking batch. Items were directly assigned to the corresponding customer’s cardboard box, leveraging higher picking density to reduce average travel time per order.

Subjects were free to determine their route strategies and were allowed to process multiple order lines with different items simultaneously. Additionally, these scenarios were conducted without any disruptions or errors in the picking lists—representing a “perfect run”. In scenarios 7 and 8, only one subject worked in the laboratory to circumvent waiting times. All eight scenarios are summarized in Table 3.

#### 3.1.3. Sensor Configuration

**Figure 7:**
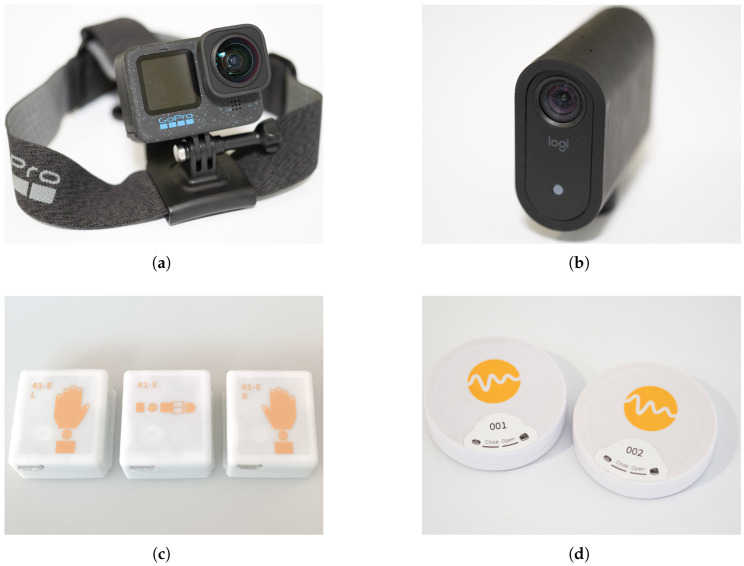
Sensors used to capture movements of the subjects and picking carts: (**a**) GoPro 12 action camera with ultra-wide-angle digital lens and head strap, (**b**) Mevo camera from Logitech without tripod, (**c**) one wearable set from MotionMiners with IMU and BLE sensors in each of the three devices, (**d**) first two beacons from MotionMiners.

The logistics scenarios were recorded using action cameras, fixed cameras, wearable devices with IMUs and BLE Received Signal Strength Indicator (RSSI) sensors, and Beacons (see Figure 7). Additionally, a cloud-based warehouse management system from Logistics Reply (https://www.reply.com/, accessed on 18 January 2026) logged the picking activities using a PDT.

**Action Cameras**

Each subject was equipped with a GoPro Hero 12 action camera (https://gopro.com/, accessed on 18 January 2026) for first-person view (FPV). The camera was attached to the forehead and pointed slightly downwards to capture not only the subjects’ field of vision but also the movements of their arms and legs. Due to individual adjustments, the viewing angles varied slightly.

**Figure 8:**
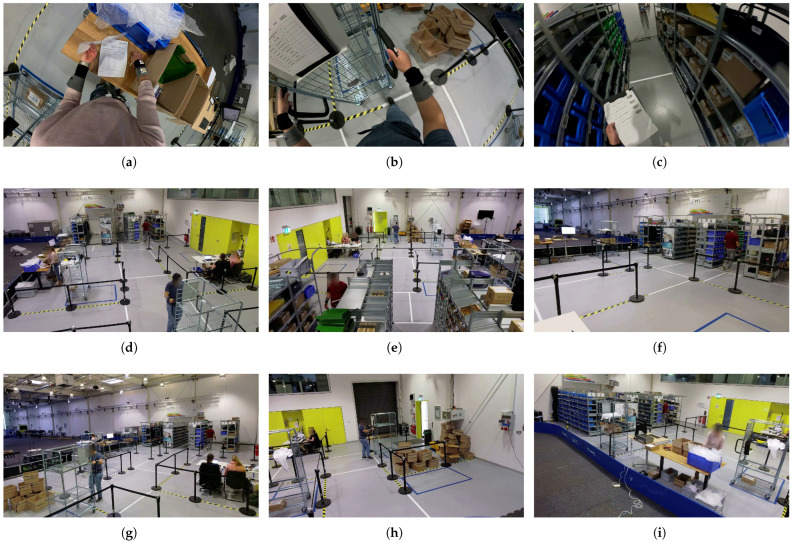
The images show the synchronized perspectives of all nine cameras at frame 31,171 in recording session 5. (**a**–**c**) display the first-person views of the subjects captured by action cameras: (**a**) Subject S13 is located in the *Packaging/Sorting Area*, (**b**) Subject S14 is on the *Path* in front of the *Cardboard Box Area*, and (**c**) Subject S15 is located in the front of *Aisle Path 4*. (**d**–**i**) show the six fixed cameras, numbered according to the dataset. (**d**) Fixed Camera 1: Main camera for annotation placed in *Cardboard Box Area*, directed towards the aisles and Fixed Camera 2. (**e**) Fixed Camera 2: Positioned in *Aisle 3*, facing Fixed Camera 1. (**f**) Fixed Camera 3: Located in the *Office*. (**g**) Fixed Camera 4: Placed in the goods *Issuing/Receiving Area*. (**h**) Fixed Camera 5: Facing the goods *Issuing/Receiving Area* and the hall gate. (**i**) Fixed Camera 6: Oriented towards the *Packaging/Sorting Area* and *Aisle 1*. The position of the fixed cameras is also shown in Figure 9 of the floor plan.

The ultra-wide-angle digital lens with a field of view of up to 177° allowed the largest possible recording field to be covered (see Figure 8a–c). The FPV videos were used for documentation, annotation, and revision.

**Figure 9:**
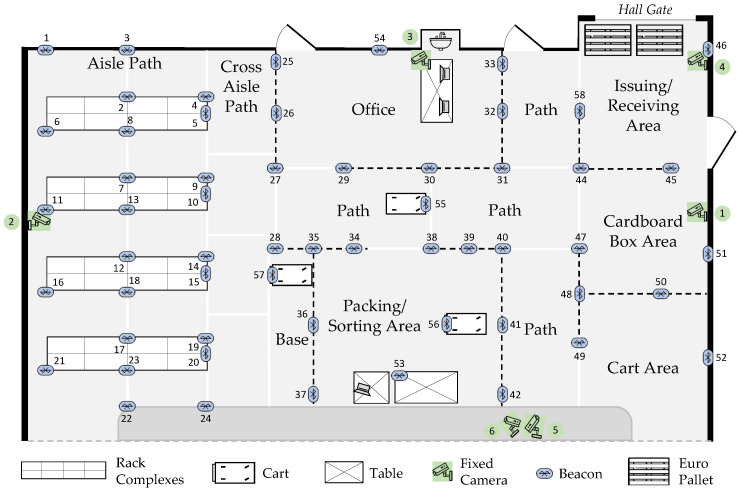
Floor plan of the Picking Lab: positions of the six fixed cameras (green) and the 57 beacons (blue). Beacons 1–42, 44–54 and 58 are stationary, while beacons 55–57 are dynamic and attached to the picking carts. (Beacon 43 was defective, so it was not used).

Mounting the camera on the forehead proved to be the optimal solution for comprehensive field-of-view coverage and an annotation-friendly perspective, especially compared to mounting it on the shoulder or chest. Nevertheless, some limitations in use occurred during the recordings. Some subjects found the mounting pressure uncomfortable, and the camera was unstable on those with straight hair. This occasionally required the subjects to readjust the camera during recording. Subject S01, in particular, frequently corrected the camera position at the beginning as the camera had slipped.

The recording was made at 29.97 fps and was interrupted only during a battery change during the session. The battery change resulted in black sequences in the action camera videos.

 

**Fixed Cameras**

In addition to the action cameras, six permanently installed Mevo cameras from Logitech (https://mevo.com/, accessed on 18 January 2026) were utilized to capture the entire test field. These cameras enabled a third-person view (TPV) of the subjects (see Figure 8d–i). The TPV videos were also used for annotation and revision. In particular, fixed camera 1 (see Figure 8d) provided a comprehensive overview and served as the primary stream for annotation, alongside the FPV videos from the action camera. The recordings were also made at 29.97 fps and ran uninterrupted throughout the entire session, ensuring complete documentation.

 

**Wearable Devices with IMUs and BLE RSSI sensors**

The subjects’ movements were recorded using wearable sensor sets comprising three MotionMiners devices. Every device is equipped with a three-axial IMU and a BLE sensor. The three devices were attached to both wrists and to the front of the torso with a belt. Pictograms on the devices ensured correct placement to minimize attachment errors (see Figure 7c).

Each subject wore two of these sets to mitigate sensor failure and enable analysis of data quality. Throughout the entire recording session, the wearable devices remained securely attached to the subjects and were not adjusted by the study supervisors. The IMUs comprise linear acceleration and angular momentum sensors operating at a sampling rate of 100 Hz. The Bluetooth sensor measured the RSSI for all received beacon-emitter signals at a sampling rate of 10 Hz.

All sensor data are stored on the sensors during recording and are transferred upon recording completion. This approach eliminated potential disruptions from wireless data transmission and ensured a robust, interference-free data collection process without requiring intervention.

 

**Beacon Emitters**

To track the subjects’ positions, 54 Bluetooth beacons were placed evenly across the Picking Lab (see Figure 9). The beacons were placed at heights ranging from 0.7 to 1.3 m on various structures, including racks, walls, a table, and barrier stands. Additionally, a beacon was placed on each picking cart at a height of 0.9 m to enable its position to be tracked when a subject was using it.

A notable challenge in position tracking arises from the varying environmental conditions. In the *Aisles* between the racks, Bluetooth signals are physically shielded by the metal racks and stored items. This results in stronger signal attenuation, which simplifies local positioning because signals can be clearly assigned to specific areas. In contrast, Bluetooth signals propagate more evenly in open areas such as the *Office*, the *Base*, and the *Packaging/Sorting Area*. This uniform signal distribution complicates region-based tracking, making it harder to distinguish between areas and increasing the likelihood of misassignments. The tracking was achieved by a proprietary machine learning algorithm. An initial calibration for each region helps account for differences in propagation.

### 3.2. Subjects

**Figure 10:**
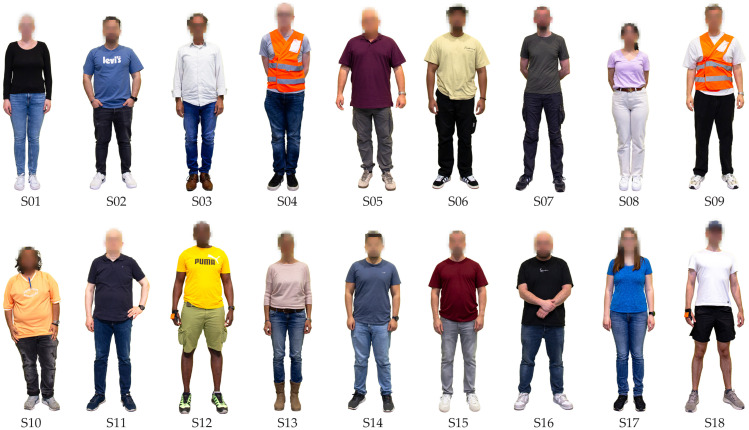
The 18 subjects of the dataset. In each recording session, three subjects took part simultaneously. To easily distinguish the subjects within a session, they wore upperwear or vests of different colors. For instance, in session 5, subjects S13, S14, and S15 wore a pink, a blue, and a red top, respectively (video footage from session 5, see Figure 8).

A total of 18 subjects participated in the data collection (see Figure 10). The selection process aimed to ensure a realistic representation of the working population in the German warehouse sector. Therefore, individuals aged up to 67 years were considered, as the statutory retirement age in Germany for those born after 1964 is 67 years. Ultimately, the sample included subjects aged 21 to 67 years, with a broad age distribution spanning individuals in their 20 s, 30 s, 40 s, 50 s, and 60 s.

According to a study by the Bundesvereinigung Logistik e.V. (BVL) from 2019, the proportion of women in the logistics, transport, and traffic sector is approximately 23%. The highest proportions of women are found in logistics-related service providers (23%) and warehousing (30%). Based on this percentage, a sample of 18 subjects would be expected to include approximately 4.14 to 5.4 women. Accordingly, five women were recruited for the study. However, in the end, only four women (22.22%) and 14 men (77.78%) participated in the study.

To ensure a diverse sample in terms of movement patterns, behaviors, and technological competence, additional demographic and physical attributes were considered. The subjects’ heights ranged from 160 cm to 187 cm, while their weights varied between 62 kg and 103 kg (see Table 4). Moreover, multiple native languages were represented, including Bengali, German, English, Greek, and Turkish. The inclusion of linguistic diversity was intentional, as warehouse environments frequently employ workers with diverse language backgrounds.

The sample included one left-handed subject. Considering that the average proportion of left-handed individuals in the general population is 10.6% [110], a slightly higher representation would have been desirable, indicating a minor sampling bias.

Subjects were not explicitly selected based on prior warehouse experience. However, seven subjects had prior experience in order picking, while 12 subjects had experience in packaging, gained through apprenticeships, internships, or part-time jobs. One of these subjects (S01) had full-time work experience as an industrial clerk. Additionally, three subjects had previously participated in a similar study (LARa dataset [10]). All 18 subjects had a University Entrance Qualification and either held a university degree or were in the process of obtaining one at the time of the study. The potential influence of educational background on movement patterns and behaviors was not explicitly analyzed in this study, as its impact was assumed to be negligible.

### 3.3. Data Recording

The data recording process is divided into three distinct phases. First, preliminary trial runs are conducted. Second, the recording process is conducted in accordance with the scenarios defined in Section 3.1.2. Third, the results of the recording are analyzed in terms of their scope and subject-specific conspicuities.

#### 3.3.1. Preliminaries

Several days prior to data recording, subjects completed an online questionnaire and received detailed study information, along with the informed consent form. The *Subject Information and Consent Form* is part of the dataset available on Zenodo. On the day of data recording, the subjects had the opportunity to clarify any open questions with the study coordinator.

Before the actual recording, all three subjects in a session completed a trial run. During this phase, they were introduced to three information technologies (picking list, glove scanner, and PDT) and the complete order picking process. For training purposes, they processed three picking orders, each consisting of three order lines (different items). This trial run was solely intended for familiarization with the processes and technologies and was not recorded. Afterward, subjects were equipped with IMUs and an action camera, marking the start of data recording.

#### 3.3.2. Recording Process

To guarantee a natural movement flow and authentic behavior of the subjects, the study supervisors were available to answer questions after the trial run, but provided minimal instructions.

At the commencement of each session, subjects performed up to three synchronization movements in the *Office*. These included the convergence of the extended arms above the head, the convergence of the extended arms in front of the chest, and the execution of a jump. These movements were subsequently utilized for synchronizing video data with the wearable sensor data.

**Figure 11:**
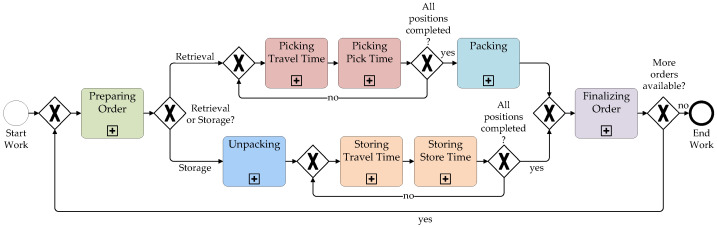
Idealized Business Process Model and Notation (BPMN) of the high-level processes *Retrieval* (upper path) and *Storage* (lower path) with its mid-level processes.

**Retrieval:** Following the synchronization, the retrieval process was initiated. Each subject was tasked with working through scenarios 1–3 (see Figure 11 and Section 3.1.2). The paper-based picking lists were available in sufficient quantities, ensuring no delays when retrieving a new order. Conversely, only one PDT and one glove scanner were available, which occasionally led to waiting times in the office as subjects had to wait until others had completed their picking and returned the hardware. Due to the shared use of the PDT and the glove scanner, scenarios 2 and 3 were never conducted in parallel, resulting in variations in the sequence of the first three scenarios across subjects. The sequence of all scenarios is available in the documentation file on Zenodo.

**Storage:** Following the completion of the initial three retrieval scenarios, each of the three different customer orders (ID 2904, 2905, 2906) was available three times in the *Issuing/Receiving Area*. Each subject was then assigned a customer order, which they unpacked and stored three times (see Figure 11).

In four of the six sessions, one subject (S01, S04, S09, and S14) simultaneously retrieved two orders (scenario 7) and subsequently stored them again (scenario 8).

After each session, subjects performed up to three synchronization movements again.

The workflows of the high-level processes *Retrieval* and *Storage*, as described and illustrated in Figure 11, represent an idealized model. During data collection, occasional deviations occurred. A more detailed visualization of the mid-level processes from Figure 11 is provided in Appendix A.

#### 3.3.3. Recording Results

The data collection took place over three days, with six sessions (two per day). In each session, three subjects participated simultaneously in the Picking Lab (see Table 5). In total, data from 18 subjects were recorded over 31:55:26 h (hh:mm:ss). The individual recording durations varied between 01:20:41 and 02:35:11 h, depending on factors such as processing speed, technological competence, performed scenarios, unintended errors, waiting time, habituation effect, and fatigue.

**Processing Speed**: The durations indicate a moderate age-related increase in the time required. Subjects in the older age group (≥50 years) required up to approximately 35% more time than the youngest subjects (≤30 years). Although without prior experience, Subject S14 (32 years) demonstrated a significantly higher processing speed compared to the average. Across all scenarios, S14 completed tasks faster than the group mean: 36% faster in scenario 1, 23% faster in scenario 2, and 17% faster in scenario 3. In scenarios 1 and 2, S14 not only achieved the fastest completion times but also executed the tasks without picking errors. The two slowest subjects, S15 and S12, were 43 years old at the time of data recording. Weight shows a slight positive relationship with processing time, whereas height and gender appear to have no relevant influence.

**Technological competence**: Subject S14 demonstrated strong performance in operating the PDT in scenario 2. Despite having no prior experience with the device, S14 adapted quickly and performed significantly better than most subjects. In contrast, subject S11 encountered considerable difficulties in using the PDT, requiring 00:34:10 h to complete scenario 2, 63% slower than the average. Due to time constraints, S11 was unable to proceed with scenario 3.

**Performed scenarios**: It was planned that each subject would go through retrieval scenarios 1 to 3, as well as a storage task from scenario 4, 5, or 6. Four of the eighteen subjects additionally completed the storage and retrieval scenarios 7 and 8, resulting in longer overall recording durations for these subjects.

**Unintended errors**: Certain subjects made unintended errors that affected their processing times. For instance, S03 and S11 overlooked the second and/or third pages of the picking list, resulting in incomplete picking and packing. This resulted in shortened processing times for S03 in scenario 3 (00:09:34 h) and S11 in scenario 1 (00:17:13 h).

**Waiting time**: The category *Other* (see Table 5) accounts for waiting times between scenarios, as well as preparatory and follow-up activities at the beginning and end of each recording session. During the packing process, subjects were instructed to wait whenever another subject was still active in the packing area. They also had to wait if the required IT was being used by someone else. This resulted in intentionally induced waiting times of up to several minutes (e.g., subject S14 with 00:35:15 h).

**Habituation effect**: After the initial execution of the scenarios, a habituation effect was observed. Subjects’ workflows appeared smoother, and the time required for similar, recurring tasks decreased in both storage and retrieval processes. In scenario 8, subjects required approximately 60% less time for storage compared to their previous scenarios 4–6. Although the execution time for retrieval increased by about 24% (from scenarios 1–2 to scenario 7), the number of positions to be picked and packed simultaneously doubled, indicating an adaptation to increased task complexity.

**Fatigue**: During data recording, some subjects exhibited signs of fatigue, which were reflected in their scenario completion times. While subject S14 recorded the fastest times in scenarios 1–3, a noticeable decline in performance was observed in scenarios 7 and 8. Compared to the other subjects, S14 was the slowest in both cases, requiring 22% more time than the next slowest subject in scenario 8. These findings suggest that fatigue may have resulted from extended recording sessions due to waiting times and the high work pace maintained during the initial scenarios.

### 3.4. Class Categories and Class Labels

Prior to annotation, 12 class categories (CC01–CC12) were defined to describe the execution of the scenarios. These categories are divided into human movements and contextual information. An overview of the class categories and class labels is provided in Table 6. Detailed label descriptions and examples are available in the documentation file on Zenodo.

The first five categories capture human movements, ranging from the *Main Activity* (CC01) to four *Sub-Activities* (*Legs*, *Torso*, *Left Hand*, and *Right Hand*). *Sub-Activities* can be regarded as semantic descriptions of a *Main Activity* but also exist independently. In the literature, such semantic descriptions are commonly referred to as attributes, detailed postures, current actions, or atomic actions [111].

Categories CC06–CC12 capture contextual information, which refers to complementary information that places an activity within its content-related, procedural, and spatial frames. This includes the customer *Order* (CC06), the use of *Information Technology* (CC07), the embedding of the activity in *Processes* (CC08–CC10), and the *Location* of the subject (CC11) or the picking cart (CC12).

Certain categories are organized hierarchically. *Sub-Activity–Left Hand* and *Sub-Activity–Right Hand* are subdivided into *Primary Position*, *Type of Movement*, *Object*, and *Tool*, while *Locations* is subdivided into *Main Area*, *Path*, *Cross Aisle Path*, *Aisle Path*, and *Other*.

Depending on the category, between four and 35 class labels were defined, yielding a total of 207 distinct labels. When all categories are combined, annotation and revision result in 68,174 unique label representations. A total of 3,444,327 frames were annotated and revised for each of the 12 class categories. The label *Unknown*, used when annotators were unsure, was largely resolved during review and remains in only 0–0.07% of cases. It does not occur in CC06–CC09, CC11, or CC12 and appears in less than 0.01% (CC10) to 0.07% (CC05) of the remaining classes.

**Figure 12:**
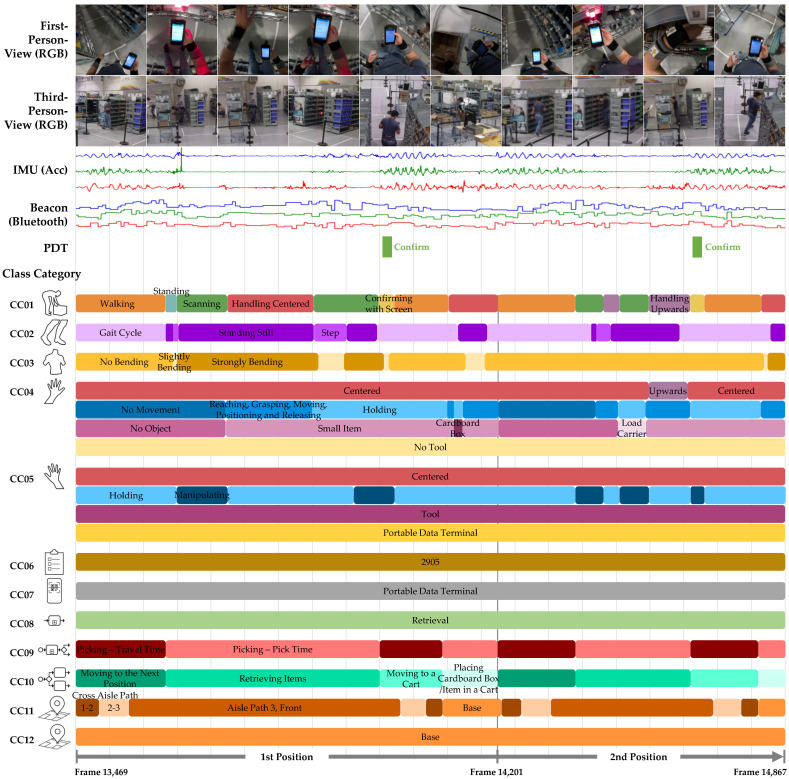
Example sequence from the DaRA dataset (duration: 1398 frames ≈46.6 s) showing two order picking positions performed by subject S14 during the scenario 2 of the recording session 5. The first four layers display sensor data, including cropped first-person views and examples from five of the six third-person RGB camera views, energy of the inertial recordings from the MotionMiners IMU set 44-C (blue = right wrist, green = belt, red = left wrist), and RSSI from the same device set connecting to the beacon number 13 (position of beacon see Figure 9). The fifth layer shows two pick confirmations transmitted by PDT and stored in the WMS. The subsequent layers depict the 12 class categories with their revised labels, where one label of some categories (e.g., *CC06 Order*; see Table 6) spans the entire sequence, while others (e.g., *CC02 Sub-Activity - Legs*) contain multiple annotation segments with different labels. (The style of this figure is based on [6]). A video of this sequence is available on YouTube (https://youtu.be/qU0XvKY20SE, accessed on 18 January 2026).

Figure 12 illustrates the complexity of the annotation process and the resulting label representations. The example depicts the order picking process for two positions (order lines), each comprising the route from the base to the item, retrieval of the item from a rack, scanning the barcodes of the storage compartment and the item, confirmation of the retrieval, transportation of the item back to the base, and placement on the picking cart.

In the first frames of Figure 12, the picker begins processing the first position by walking along the cross aisle to the next position in the third aisle path, holding a portable data terminal in the right hand, with the left hand inactive, performing the retrieval process (order 2905) during the travel time phase, while the cart remains at the base.

Upon reaching the rack, the subject stands and searches for the correct compartment, checking the display for the quantity to be retrieved. The *Strongly Bending* motion then begins as the subject scans the storage location code. The onset of the bending movement is clearly visible in the torso IMU data (green) as a distinct peak (see Figure 12). Additional motion segments and their corresponding labels can also be visually identified in the IMU signals—for instance, those belonging to class CC02. Segments of the *Gait Cycle* are characterized by rhythmic oscillations in the torso IMU data (green), whereas the signal amplitude becomes more erratic and decreases noticeably during *Standing Still* and *Step* phases.

### 3.5. Annotation and Revision

The following sections describe the methodology, procedure, and time effort involved in annotation and revision.

#### 3.5.1. Annotation Methodology

After video export and synchronization, annotation and subsequent revision—following the *silver standard with more-experienced revisers* as described in [112]—were conducted using the SARA tool [88,109]. The labels are annotated segment-wise in time, meaning that label segments were defined with flexible durations based on the natural onsets and offsets of movements. The commencement and cessation of these segments, in addition to the selection of labels, are determined by the annotator’s perspective.

Regarding label exclusivity, either a single label or a multi-label was applied, depending on the class category. With a single label, exactly one label was assigned to each time interval, as in class categories CC01, CC02, CC07, CC08, CC09, and CC10 (for class categories, see Table 6). In contrast, in the multi-label category, either multiple labels were allowed (CC03, CC06, CC11, CC12) or required (CC04, CC05).

All labels were assigned as hard labels, i.e., with unambiguous allocation (e.g., 100% *Walking* in CC01 or 100% *No Bending* in CC03; see the example in Figure 12).

#### 3.5.2. Annotation Sessions

The dataset was manually annotated by 15 domain experts and by trained internal annotators. Each annotator received instructions and an annotation guideline (see the *Documentation.pdf* file) and performed test annotations (see [113]) to ensure the label quality. Subsequently, annotators were assigned specific subjects, and each recording was annotated in full by exactly one annotator per class category (single-annotator labeling).

Annotation was performed either individually or jointly, depending on the class category (see Table 6). *Main Activity* (CC01) and *Locations* (CC11–CC12) were annotated separately, while CC06–CC08 and CC09–CC10 were annotated jointly. A sequential annotation strategy was applied to reduce effort and minimize errors, particularly for less experienced annotators.

To further increase consistency, we integrated dependency rules into the tool as CSV files that specify all valid label combinations within and across categories. Once this dependency file is imported, the annotator is prevented from selecting any invalid combinations. For example, *Walking* and *Standing* cannot co-occur in *Main Activity*. Similarly, *Sub-Activities* (CC02–CC05) were annotated with reference to the previously assigned *Main Activity*, ensuring segment alignment. In this way, annotators of *Sub-Activities* simultaneously acted as revisers of *Main Activity*.

#### 3.5.3. Annotation Revision

**Figure 13:**
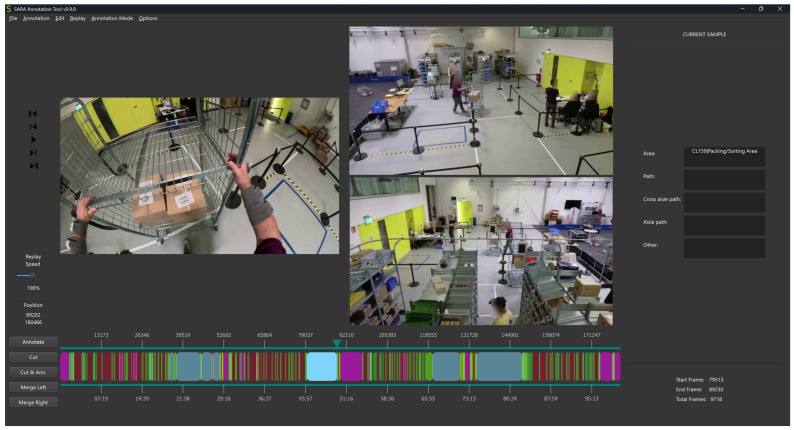
Screenshot from the tool SARA [88,109], displaying the fully annotated and revised recording (180,466 frames) of the *Location–Human* class category for subject S05 wearing a purple shirt during session 2. On the left, the FPV from the action camera is shown, while the right side displays the TPV from fixed cameras 1 and 2. To the right of the videos, the annotator assigned the label *Packaging/Sorting Area*, which was verified by the reviser. The lower color gradient represents the set segments. Each of the 564 segments corresponds to a new area, with the segment width indicating the duration the subject remained in that area. The colors align with the floor plan coding scheme (see Figure 4). The displayed frame (89,202) captures the subject in the *Packaging/Sorting Area* (light blue), just as they are about to leave it and enter the *Path* (lime green).

Following the annotation, both manual and automated revision of the annotated labels were conducted. During manual revision, each annotated file (e.g., *Location–Human* class category for subject S05; see Figure 13) was imported into SARA Tools with the video files, thereby enabling synchronized playback of both the videos and the labels. Each file was revised once (except files from *Main Activity*) by one of the eight revisers. In cases of a mislabeled or misplaced segment, new labels were assigned, or the start and end boundaries of segments were adjusted.

The *Main Activity*, unlike the other class categories, was revised not separately but in parallel with the annotation of the *Sub-Activities* CC02–CC05. For this purpose, the already annotated class labels of the Main Activity were extended by the class labels of CC02. The goal was to preserve the existing Main Activity segments, as they are conceptually closely related to the Sub-Activities. During the annotation of CC02, the annotator examined the Main Activity and manually corrected it when necessary. Due to the use of dependencies, the incorrectly annotated Main Activity labels had to be adjusted to assign the CC02 class labels. For example, it was not possible to annotate *CL016|Gait Cycle* if *CL012|Standing* was already assigned within the same segment. This procedure was repeated analogously for CC03 through CC05, with the revised Main Activity labels carried forward continuously.

In addition, automated plausibility checks were applied to identify frames with an excessive or insufficient number of labels (the number varies depending on the class category), as well as mutually exclusive label sequences either within a single class category (e.g., in the class category *Location*, the label *Aisle Path* cannot directly follow *Cart Area* because other areas are in between) or across multiple categories.

#### 3.5.4. Time Effort

For all class categories, the annotation required 1572 person-hours (PH), and the revision amounted to 361 PH (see Table 7). The time requirements refer exclusively to the main annotation and revision. They do not include time spent on annotator training, test annotations, or correcting errors identified through automated checks. The effort varied significantly across class categories. In the case of annotation, the average labeling effort for a one-minute video ranged from 6 sec (*Order*, *Information Technology*, and *High-Level Process*) to over 12 min (*Sub-Activity–Left Hand*).

### 3.6. Available Data and Dataset Utilization

The DaRA dataset, available through the Zenodo repository [15], supports a broad spectrum of research applications such as human activity recognition, human context recognition, indoor localization, process mining, and process recognition.

The dataset comprises both **sensor data** and **revised annotations** for three sensor modalities: camera (29.97 fps), IMU (100 Hz), and BLE (10 Hz). Within each recording session, the three FPV and six TPV camera streams are temporally synchronized, i.e., they start simultaneously but may differ in overall duration depending on the speed at which the recording session is completed. In total, the FPV camera recordings comprise 31:55:26 h (see Table 5) and the TPV camera recordings amount to 77:18:24 h. Video annotations were synchronized with the wearable sets (IMU and Beacon) based on defined synchronization movements. The **Python script** (version 1) used to perform the synchronization is available on GitHub [106]. Further details are provided in the *Documentation.pdf* file. In total, the dataset contains 1056 revised annotation files, including the following:

- A total of 216 annotations for the synchronized cameras (18 subjects × 12 class categories);
- A total of 420 annotations each for the IMU data and the Beacon data ([18 subjects × 2 wearable sets—one faulty wearable set from subject S10] × 12 class categories).

The **WMS data** (available in CSV format) includes picking confirmations recorded via the PDT. All timestamps are synchronized with the video data and the corresponding annotation files.

Class label configuration files, called **scheme files** (available in JSON format), enable the import, visualization, and editing of annotations in conjunction with the video recordings within the SARA annotation tool [88,109]. A dedicated scheme file is provided for each class category, defining the corresponding annotation structure.

The accompanying **documentation file** (PDF format) provides further information on the dataset. It includes an annotation guideline, detailed descriptions of all 12 class categories and their 207 class labels, and the master item data, containing information on storage location, physical dimensions, and weight. Furthermore, the documentation features item photographs, the three customer orders, and information about the sensor placement, synchronization, and sequence, as well as the start and end of the scenarios.

Finally, a **Python script** for **preprocessing** (ZIP archive) is provided to enable customized modification of the annotation files [106]. The script supports interactive selection of class categories, with optional filtering of *Unknown* and/or *Other* labels. It decomposes structured classes by splitting *Location—Human/Cart* into Main and Sub locations, and *Left/Right Hand* into *Primary Position*, *Type of Movement*, *Object*, and *Tool*. It can also construct compact *input* and *output* combination columns for downstream analysis. The processed results are exported as synchronized per-subject CSV files.

## 4. Evaluation—Dataset Quality

The overall quality of the dataset is determined by the consistency of the labels after annotation and revision, the quality of the acquired sensor data, and the application of the labels and sensor data for HAR.

### 4.1. Annotation and Revision Quality

The annotators were required to conduct test annotations for each class category they annotated or revised. For the class categories CC06–CC08, the entire recording of subject S09 was test-annotated. For the other categories, excerpts of over five minutes from subjects S04, S05, and S06 were test-annotated. The revisers had to revise the test annotations for their respective class categories. The test video recordings, along with all test annotations and revisions, have been published as an additional dataset on Zenodo [113].

To assess annotation quality as well as the revised datasets, we used Cohen’s κ [114] for exactly two annotators and Light’s κ [115] for more than two annotators, the latter being the mean of all pairwise Cohen’s κ values. Overall agreement was summarized as macro-κ, defined as the unweighted average of per-label κ. Because extremely rare labels in the test annotations can yield unstable κ estimates (e.g., if only one annotator marked 10 frames in the test annotation as *Sitting* while others did not), we pre-specified a filtered macro-κ that includes only labels with sufficient support (≥0.5% of frames and ≥30 positive frames).

After revision, we observed high average macro Light’s κ across categories, ranging from 78.27% (CC02, *Sub-Activity—Legs*) to 99.88% (CC06, *Order*) (see Table 8). According to common benchmarks [116,117,118], this corresponds to *substantial* to *almost perfect* agreement. We also found a clear difference between human movement categories (CC01–CC05; 78.27–81.61%) and context categories (CC06–CC12; 90.95–99.88%). A similar pattern is already visible in the unrevised test annotations. Overall, these findings indicate that context segments were easier and more consistently labeled than human movements.

Significant enhancements from the annotation to the revision, for example, in CC02 (*Sub-Activity—Legs*; from 60.99% to 78.27%), in CC03 (*Sub-Activity—Torso*; from 40.83% to 81.61%) and CC10 (*Low-Level Process*; from 73.25% to 90.95%), are partly attributable to the use of *Another* and *Unknown* labels during annotation whenever an unambiguous assignment seemed infeasible. During revision, such segments were typically reassigned to more specific labels, thereby substantially increasing agreement.

### 4.2. Sensor Data Quality

The recordings from all six fixed cameras are synchronized and corrected across all six sessions. Correcting here means adding blank frames to keep all the videos synchronized for annotation purposes. Due to a battery change, the cameras did not record the same material. However, synchronization and data integrity are unaffected. All nine video streams from the action and fixed cameras were automatically synchronized and then manually verified and corrected as needed. The synchronization of the videos for each session was verified using several sections with rapid movements by the subjects, such as gait cycle, and white markings on the floor. Any residual temporal offsets are minimal, on the order of zero to three frames. An illustrative example is shown in Figure 8, where a one-frame offset is visible: in (b) the foot remains on the line, whereas in (h) it has moved slightly behind it.

The MotionMiners devices record IMU data and RSSI from all the beacons in the layout. Each device set per subject comprises three devices (right arm, left arm, and torso) and records a three-dimensional accelerometer, a three-dimensional gyroscope, and RSSI readings from all beacons spread across the layout. The RSSI signals are used for indoor localization by means of a fingerprinting method, where a region is represented by statistical features from the RSSI signals from the three devices for a specific period of time. Localization is carried out by distance classification.

The MotionMiners devices guarantee the recording of IMU data with no data loss at a sampling rate of 100 Hz, as they record the data and transfer it upon completion. Still, when devices are damaged, complete recordings are lost—MotionMiners seeks to reduce such cases. One of the two device sets of test subject S10 recorded incorrectly and is therefore not included in the dataset.

### 4.3. Quality of Revised Annotations and Sensors Combined—Deploying DaRA for HAR

We trained a tCNN-IMU, similar to [4,10], using the IMU data as a HAR baseline. This serves as a high-quality example showing that the data and annotations can, in principle, be used to train AI methods. The tCNN-IMU processes sequence segments with a feature map input of size [T,18], where *T* is the sequence length and 18 is the number of sequence channels, corresponding to [x,y,z] accelerometer and gyroscope measurements from the three devices. The sequence segments are extracted following a sliding-window approach with a window size of T=150, step size of s=25 (16.7% overlapping). The tCNN-IMU computes either an activity class k or a binary-attribute representation a. An attribute representation is a combination of sub-activity labels (short activities or limb movements) a∈B, creating a sort of semantic description of an activity. Each attribute indicates whether a specific sub-activity is present during the activity. Following [4], input sequences are normalized per sensor channel to the range of [0,1]. Additionally, a Gaussian noise with parameters [μ=0,σ=0.01] is added.

Following the training procedures from [3,42], the IMU data is divided into three sets: training, validation, and testing. The training set comprises recordings from subjects [S02,S03,S04,S06,S07,S08,S10,S11,S12,S13,S15,S16]. The validation and testing sets are composed of recordings from [S01,S05,S18] and [S09,S14,S17], respectively. An early stopping approach is followed using the validation set. This set is also used to find appropriate training hyperparameters. Recordings with labels *Synchronization*, *Another Main Activity*, and *Main Activity Unknown* are not considered for training. The architecture is trained using batch gradient descent with RMSProp, with an RMS decay of 0.9, a learning rate of $1\times 10^{-4}$, and a batch size of 400. Moreover, Dropout was applied to the first and second fully connected layers. The tCNN-IMU is trained using a softmax layer to predict activity classes directly with Cross-Entropy Loss, or a Sigmoid layer to predict an attribute representation with Binary-Cross-Entropy Loss.

Table 9 and Table 10 present the performance of the method solving HAR on the DaRA IMU dataset using the softmax layer and sigmoid layer. Precision is computed as $P=\frac{TP}{TP+FP}$. Recall is computed as $R=\frac{TP}{TP+FN}$. Having TP, FP, and FN as the true positives, false positives, and false negatives. The weighted F1 is calculated as $wF1=\sum_{i}^{C}2\times \frac{n_{i}}{N}\times \frac{P_{i}\times R_{i}}{P_{i}+R_{i}}$, with $n_{i}$ being the number of window samples of class $C_{i}\in C$. *Confirm with Pen*, *Walking* and *Standing* activities show the best performances. These results align with [4,9], which show that using attribute predictions for HAR improves classification performance. However, these are preliminary results, as the DaRA datasets include multiple annotation levels; HAR and process predictions using HMMs, transformers, or LSTMs should be considered.

Table 11 shows the confusion matrix from the activity class predictions using the tCNN-IMU with the softmax layer. The three *confirm* activities show poor performance, i.e., low precision with very high false positives. These activities are very difficult because they have a shorter duration than others, e.g., with fewer samples. Besides, these activities are not carried out by all the subjects. *Scan* tends to be predicted as *Handling Center*, which are semantically similar.

We primarily experimented with IMU data for human activity recognition (HAR) using main activity labels and sub-activity labels with an existing method, namely tCNN-IMU [4,10]. This initial evaluation of DaRA provides a baseline for HAR. Process prediction, localization using BLE RSSI, and the combination of multiple devices and label types are to be carried out as part of future work. As part of future work, we aim to experiment with the relationship between activities and location using low- and mid-level processes, using learning methods such as LSTMs, Transformers, and HMMs. This experimentation is based on the strong relation between repetitive activities, location areas, and structured processes within logistics tasks.

## 5. Discussion and Future Works

### 5.1. Discussion

This paper introduced the DaRA dataset, a novel multimodal dataset for HAR and HCR in intralogistics. It includes multiple sensors and extensive class labels that describe both human movements and context, allowing activities to be characterized in terms of content, procedure, and spatial setting.

The DaRA dataset helps address key research gaps in HAR and HCR. First, there is a lack of datasets specifically designed for industrial domains. Second, existing datasets often lack contextual sensor data or labels, which are essential for a comprehensive understanding of activities. Finally, DaRA offers rich metadata that are rarely available in comparable datasets.

The dataset provides high-quality annotations and detailed sensor data. Limitations arise from the recording environment and subject characteristics. The semi-controlled lab setting enables realistic movements but does not fully reflect real-world warehouse processes, and only selected intralogistics processes and technologies are covered. Furthermore, women are underrepresented among subjects, and the subjects were not professional warehouse workers.

A trained neural network achieved an F1 score of over 73.70% for activity recognition, demonstrating successful classification of human movements. The next logical step is to advance HCR, where context may be derived from both sensor data and classified activities.

### 5.2. Future Works

The created DaRA dataset can be used for the well-established field of HAR as well as for HCR. HCR encompasses indoor localization and, in particular, the still underexplored and increasingly relevant research area of process recognition. For a comprehensive optimization of workflows in a warehouse environment, it is not sufficient to know solely *what* a person is doing (main and sub-activity); it is equally important to determine *where* (location) and, most importantly, *within which process step* this activity is being performed. In this way, recognized activities can be embedded into a semantically meaningful and human-interpretable context.

Furthermore, the dataset offers substantial potential for logistics simulations, motion prediction, policy learning in robotics [119,120], multi-view integration, the detection and identification of logistics objects, and studies on RGB-based person and action recognition. Although multimodal data have been shown to yield superior predictive performance in neural networks compared to unimodal approaches, DaRA deliberately pursues the objective of enabling robust recognition using as few information sources and sensor types as possible in industrial settings. Consequently, a unimodal design was adopted in this work to produce a lightweight neural network architecture that operates reliably with reduced GPU resources. Nevertheless, future experiments incorporating multimodal data are desirable to systematically evaluate the trade-off between additional sensor modalities and potential gains in predictive performance.

Based on the provided labels for the recordings, further studies can be conducted on temporal jitter in motion annotations and on the derivation of textual annotations. Future versions of DaRA are also intended to provide skeletal information extracted from RGB videos, thereby facilitating policy learning in robotics and supporting simulation-based research.

## Figures and Tables

**Table 1:** Overview of the DaRA dataset (BPMN = Business Process Model and Notation, IMUs = Inertial Measurement Units).

**General**	Download DaRA Dataset	[15]
	Recording Environment	semi-controlled laboratory (Section 3.1.1)
	Scenario	warehousing: order picking, packaging, unpacking, storage (Section 3.1.2)
		BPMN (Section 3.3.2)
	Dataset Size	31:55:26 h of recording time (Section 3.3.3)
	Data Availability/Usage	Section 3.6
**Sensor** (Section 3.1.3)	Action Cameras	1 camera per subject, 29.97 fps, 32 h
	Fixed Cameras	6 cameras, 29.97 fps, 77 h
	IMUs	6 IMUs per subject (2 sets), 100 Hz
	Beacons	57 beacons, 10 Hz
**Subjects** (Section 3.2)	Number	18 (4 female, 14 male)
	Age	21 to 67 years (avg. 37.4 years)
	Weight	62 to 103 kg (avg. 81.1 kg)
	Height	160 to 187 cm (avg. 175.8 cm)
**Annotation**	Class Categories (Section 3.4)	12 categories with human movements and context
	Class Labels (Section 3.4)	207 labels, 68,174 label representations
	Annotation (Section 3.5.2)	1572 h manual annotated by 15 domain experts and trained internal annotators
	Revision (Section 3.5.3)	361 h manual revision by 8 experts and automated plausibility checks
	Label Quality (Section 4.1)	Light’s Kappa from 78.27% to 99.88% depending on the class category

**Table 2:** Overview of HAR datasets with application domains in industry (production and logistics). Note that the abbreviation *MoCap* refers to the motion capture system, *RGB* refers to colored videos, and *RGB-D* refers to colored videos along with depth information. Columns where the information is unclear or wasn’t obtained are marked with a ‘-’. ✓ indicates that the dataset is publicly available. Reference is abbreviated as Ref. and Number as Nr.

Dataset					Sensors		Subjects	Recording	Labels		Annotation
Name	Ref.	Year	Public	Size	Nr.	Type	Nr.	Environment	Category	Nr. and Type	
MPP Dataset	[64,65]	2025	✓	3:23 h	2	inertial	4	real-world	human–object interactions	7 activity classes	domain expert
IHAD_v_ 1	[66]	2023	-	459,180 images	1	visual (RGB)	-	controlled	human–object interaction	12 activity classes	not mentioned
HRI30	[67,68]	2022	✓	15 GB	1	visual	11	controlled	body pose, human–object and human–robot interactions	30 actions	manually annotated
CoAx	[69,70]	2022	✓	1:58 h	1	visual (RGB-D)	6	controlled	human–object and human–robot interactions	10 action and 8 object annotations	action and object annotation
OpenPack	[5,6]	2022	✓	53.8 h	20	visual, inertial, physiological/biosensors, other	16	controlled	human-to-object interactions	11 activity classes	expert
InHARD-DT	[71,72]	2022	✓	25.8 GB	34	visual (RGB, MoCap), inertial	12	virtual	human–object and human–robot interactions	18 event/action classes	auto-labelled
HA4M	[73,74]	2022	✓	4.1 TB	1	visual (RGB, RGB-D, Infrared)	41	controlled	human–object interaction	12 actions	manual annotation
Assembly101	[75,76]	2022	✓	513 h	13	visual	53	controlled	human-to-object interactions	1380 fine-grained, 202 coarse actions	trained annotators
COVERED	[77,78]	2022	✓	860 MB	1	visual	-	real-world	postures, human–robot interactions	6 semantic segmentation classes	
CAARL	[11,79]	2021	✓	2:33 h	46	visual (RGB, MoCap), inertial	2	controlled	postures/static activities, human-to-object interaction, locomotion	8 activity classes, 19 attributes	annotation tool SARA
WGD	[80]	2021	-	-	8	visual (MoCap, RGB)	8	controlled	posture, human–object interactions	-	-
Physical Human–Robot Contact Detection	[81,82]	2021	✓	79.9 MB	2	visual (RGB-D)	-	controlled	human–robot interactions, postures	5 actions	-
ABC Bento	[83,84]	2021	✓	499 MB	20	visual (MoCap)	4	controlled	human-to-object interaction	10 labels	participants are designing methods
InHARD	[85,86]	2020	✓	51.6 GB	35	visual (MoCap, RGB)	16	semi-controlled	human–object interaction	14 low-level, 74 high-level action classes	annotation tool Anvil
LARa	[10,87,88,89]	2020	✓	12:6 h	54	visual (RGB, MoCap), inertial	16	controlled	postures/static activities, human–object interaction, locomotion	8 activity classes, 19 attributes	annotation tool SARA
MECCANO	[90,91]	2020	✓	10.5 MB	1	visual	20	controlled	human–object interaction	61 action classes with verb and object/s and bounding box annotations	manual
IKEA ASM	[92,93]	2020	✓	35:26 h	3	visual (RGB, RGB-D)	48	controlled	human–object interaction	33 verb-object	Amazon Turk manual annotators
AndyData-lab-onePerson	[7,8]	2019	✓	5 h	31	visual (MoCap, RGB), inertial, tactile/force	13	controlled	postures/static activities, human–object interaction	6 general, 5 detailed posture, 8 action	annotation tool Anvil
PPG-DaLiA	[94,95]	2019	✓	36 h	2	inertial, physiological/biosensors	15	semi-controlled	postures/static activities, ADL, sports	9 activity labels	protocol-defined
HAD-AW	[96,97]	2018	✓	102 MB	1	inertial	16	real-world	ADL, sports	8 ADLs consisting of 31 motion primitives	not explicitly mentioned
Nath et al.	[98]	2018	-	40 min	2	inertial	2	semi-controlled, real-world	human–object interaction	5 activity labels	manually
ExtraSensory Dataset	[99,100]	2017	✓	5000 h	1	inertial, positioning, acoustic, environmental, other (phone state)	60	real-world	ADL	116 original labels, 51 cleaned labels	by the user
Skoda Mini Checkpoint	[40,101]	2008	✓	–	20	inertial	1	controlled	human–object interaction	10 gesture, 70 instances of each gesture	experimenters

**Table 3:** Specification of the eight recorded scenarios. An ‘X’ denotes that the criterion is fulfilled.

		Scenario							
		1	2	3	4	5	6	7	8
**High-Level** **Processes**	Retrieval (picking and packing)	X	X	X				X	
	Storage (unpacking and storing)				X	X	X		X
**Picking** **Strategies**	Single-order picking (serial)	X	X	X	X	X	X		
	Multi-order picking (parallel)							X	X
**Information** **Technologies**	Picking list and pen	X			X	X	X	X	X
	Portable data terminal		X						
	Picking list and glove scanner			X					
**Customer Order**	2904	X			X			X	X
	2905		X			X		X	X
	2906			X			X		
**Errors in** **Picking List**	With intentional errors	X		X					
	Without intentional errors		X		X	X	X	X	X

**Table 4:** Subject specifications. All data were collected via a digital survey (subject questionnaire) completed independently by subjects.

ID	Sex	Age	Weight	Height	Handedness	Employment	Experience [from 1 = Extensive to 6 = None]		
	[F/M]	[years]	[kg]	[cm]	[L/R]	Status	Order Picking	Packaging	Similar Studies
S01	F	32	68	171	R	Student	2	3	6
S02	M	27	76	167	R	Student	3	6	6
S03	M	64	69	171	R	Employee	6	5	5
S04	M	31	85	183	L	Employee	5	4	6
S05	M	67	100	177	R	Retiree	6	3	6
S06	M	24	82	178	R	Student	4	6	6
S07	M	41	70	180	R	Employee	6	5	6
S08	F	29	62	163	R	Student	6	6	6
S09	M	21	85	180	R	Student	6	6	6
S10	M	28	85	160	R	Student	3	3	6
S11	M	59	85	178	R	Employee	3	2	6
S12	M	43	103	186	R	Job seeker	6	6	4
S13	F	52	66	175	R	Employee	5	4	6
S14	M	32	80	176	R	Employee	6	5	5
S15	M	43	88	177	R	Employee	6	5	6
S16	M	29	100	175	R	Student	6	3	6
S17	F	25	75	180	R	Employee	6	5	6
S18	M	26	80	187	R	Student	6	6	6
Min.		21	62	160					
Avg.		37.4	81.1	175.8					
Max.		67	103	187					

**Table 5:** Subject assignment. Scope of participation in the *Scenarios*, *Other* and *Total*.

ID	Recording	Scope of the Scenarios 1–8 [hh:mm:ss]									
	Session	Retrieval (Scenario 1–3)			Storage (Scenario 4–6)			Perfect Run		Other	Total
		1	2	3	4	5	6	7	8		
S01	1	00:18:15	00:19:20	00:18:39	-	-	00:15:51	00:23:42	00:14:34	00:10:59	02:01:19
S02	1	00:19:43	00:16:36	00:22:16	-	00:23:56	-	-	-	00:15:43	01:38:14
S03	1	00:24:41	00:25:07	00:09:34	00:27:04	-	-	-	-	00:03:11	01:29:37
S04	2	00:16:22	00:16:09	00:17:57	-	00:32:17	-	00:26:00	00:14:28	00:13:17	02:16:30
S05	2	00:25:47	00:20:05	00:19:11	-	-	00:26:36	-	-	00:08:42	01:40:22
S06	2	00:22:08	00:16:45	00:17:27	00:25:27	-	-	-	-	00:02:29	01:24:16
S07	3	00:20:13	00:23:38	00:16:16	-	00:26:40	-	-	-	00:15:16	01:42:02
S08	3	00:19:47	00:20:10	00:15:49	-	-	00:21:29	-	-	00:03:57	01:21:11
S09	3	00:18:18	00:16:33	00:18:05	00:27:40	-	-	00:23:47	00:15:57	00:05:03	02:05:24
S10	4	00:25:18	00:24:02	00:21:07	-	-	00:26:50	-	-	00:13:37	01:50:54
S11	4	00:17:13	00:34:10	-	00:33:30	-	-	-	-	00:08:20	01:33:13
S12	4	00:24:24	00:26:29	00:28:18	-	00:31:17	-	-	-	00:10:33	02:01:00
S13	5	00:22:28	00:19:11	00:20:07	-	-	00:24:08	-	-	00:02:59	01:28:53
S14	5	00:13:27	00:16:07	00:15:44	00:28:18	-	-	00:26:57	00:19:23	00:35:15	02:35:11
S15	5	00:27:55	00:24:44	00:25:14	-	00:29:57	-	-	-	00:07:26	01:55:17
S16	6	00:23:11	00:17:25	00:20:22	-	-	00:20:17	-	-	00:16:24	01:37:38
S17	6	00:18:42	00:19:59	00:15:45	00:24:08	-	-	-	-	00:02:08	01:20:41
S18	6	00:20:02	00:20:53	00:20:56	-	00:37:01	-	-	-	00:14:51	01:53:43
**Min.**		00:13:27	00:16:07	00:09:34	00:24:08	00:23:56	00:15:51	00:23:42	00:14:28	00:02:08	**01:20:41**
**Avg.**		00:21:00	00:20:58	00:18:59	00:27:41	00:30:11	00:22:32	00:25:06	00:16:05	00:10:34	**01:46:25**
**Max.**		00:27:55	00:34:10	00:28:18	00:33:30	00:37:01	00:26:50	00:26:57	00:19:23	00:35:15	**02:35:11**
**Sum**		**06:17:54**	**06:17:22**	**05:22:46**	**02:46:07**	**03:01:08**	**02:15:11**	**01:40:26**	**01:04:21**	**03:10:10**	**31:55:26**

**Table 6:** Class categories and class labels (M = Human Movement, C = Context, an ‘X’ denotes that the criterion is fulfilled).

Class Categories [CC]			M	C	Class Labels [CL]	
Icon	ID	Name			Nr.	List
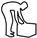	CC01	Main Activity	X		15	CL001|Synchronization; CL002|Confirming with Pen; CL003|Confirming with Screen; CL004|Confirming with Button; CL005|Scanning; CL006|Pulling Cart; CL007|Pushing Cart; CL008|Handling Upwards; CL009|Handling Centered; CL010|Handling Downwards; CL011|Walking; CL012|Standing; CL013|Sitting; CL014|Another Main Activity; CL015|Main Activity Unknown
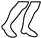	CC02	Sub-Activity–Legs	X		8	CL016|Gait Cycle; CL017|Step; CL018|Standing Still; CL019|Sitting; CL020|Squat; CL021|Lunges; CL022|Another Leg Activity; CL023|Leg Activity Unknown
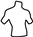	CC03	Sub-Activity–Torso	X		6	CL024|No Bending; CL025|Slightly Bending; CL026|Strongly Bending; CL027|Torso Rotation; CL028|Another Torso Activity; CL029|Torso Activity Unknown
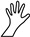	CC04	Sub-Activity–Left Hand	X		35	**Primary Position:** CL030|Upwards; CL031|Centered; CL032|Downwards; CL033|Position Unknown **Type of Movement:** CL034|Reaching, Grasping, Moving, Positioning and Releasing; CL035|Manipulating; CL036|Holding; CL037|No Movement; CL038|Another Movement; CL039|Movement Unknown **Object:** CL040|No Object; CL041|Large Item; CL042|Medium Item; CL043|Small Item; CL044|Tool; CL045|Cart; CL046|Load Carrier; CL047|Cardboard Box; CL048|On Body; CL049|Another Logistic Object; CL050|No Logistic Object; CL051|Object Unknown **Tool:** CL052|Portable Data Terminal; CL053|Glove Scanner; CL054|Plastic Bag; CL055|Picking List; CL056|Pen; CL057|Button; CL058|Computer; CL059|Bubble Wrap; CL060|Tape Dispenser; CL061|Knife; CL062|Shipping/Return Label; CL063|Elastic Band; CL064|Another Tool
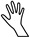	CC05	Sub-Activity–Right Hand	X		35	**Primary Position:** CL065|Upwards; CL066|Centered; CL067|Downwards; CL068|Position Unknown **Type of Movement:** CL069|Reaching, Grasping, Moving, Positioning and Releasing; CL070|Manipulating; CL071|Holding; CL072|No Movement; CL073|Another Movement; CL074|Movement Unknown **Object:** CL075|No Object; CL076|Large Item; CL077|Medium Item; CL078|Small Item; CL079|Tool; CL080|Cart; CL081|Load Carrier; CL082|Cardboard Box; CL083|On Body; CL084|Another Logistic Object; CL085|No Logistic Object; CL086|Object Unknown **Tool:** CL087|Portable Data Terminal; CL088|Glove Scanner; CL089|Plastic Bag; CL090|Picking List; CL091|Pen; CL092|Button; CL093|Computer; CL094|Bubble Wrap; CL095|Tape Dispenser; CL096|Knife; CL097|Shipping/Return Label; CL098|Elastic Band; CL099|Another Tool
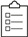	CC06	Order		X	5	CL100|2904; CL101|2905; CL102|2906; CL103|No Order; CL104|Order Unknown
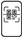	CC07	Information Technology		X	5	CL105|List and Pen; CL106|List and Glove Scanner; CL107|Portable Data Terminal; CL108|No Information Technology; CL109|Information Technology Unknown
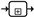	CC08	High-Level Process		X	4	CL110|Retrieval; CL111|Storage; CL112|Another High-Level Process; CL113|High-Level Process Unknown
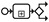	CC09	Mid-Level Process		X	10	CL114|Preparing Order; CL115|Picking–Travel Time; CL116|Picking–Pick Time; CL117|Unpacking; CL118|Packing; CL119|Storing–Travel Time; CL120|Storing–Store Time; CL121|Finalizing Order; CL122|Another Mid-Level Process; CL123|Mid-Level Process Unknown
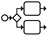	CC10	Low-Level Process		X	31	CL124|Collecting Order and Hardware; CL125|Collecting Cart; CL126|Collecting Empty Cardboard Boxes; CL127|Collecting Packed Cardboard Boxes; CL128|Transporting a Cart to the Base; CL129|Transporting to the Packaging/Sorting Area; CL130|Handing Over Packed Cardboard Boxes; CL131|Returning Empty Cardboard Boxes; CL132|Returning Cart; CL133|Returning Hardware; CL134|Waiting; CL135|Reporting and Clarifying the Incident; CL136|Removing Cardboard Box/Item from the Cart; CL137|Moving to the Next Position; CL138|Placing Items on a Rack; CL139|Retrieving Items; CL140|Moving to a Cart; CL141|Placing Cardboard Box/Item on a Table; CL142|Opening Cardboard Box; CL143|Disposing of Filling Material or Shipping Label; CL144|Sorting; CL145|Filling Cardboard Box with Filling Material; CL146|Printing Shipping Label and Return Slip; CL147|Preparing or Adding Return Label; CL148|Attaching Shipping Label; CL149|Removing Elastic Band; CL150|Sealing Cardboard Box; CL151|Placing Cardboard Box/Item in a Cart; CL152|Tying Elastic Band Around Cardboard; CL153|Another Low-Level Process; CL154|Low-Level Process Unknown
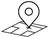	CC11	Location–Human		X	26	**Main Area:** CL155|Office; CL156|Cart Area; CL157|Cardboard Box Area; CL158|Base; CL159|Packing/Sorting Area; CL160|Issuing/Receiving Area; CL161|Path; CL162|Cross Aisle Path; CL163|Aisle Path **Path:** CL164|Path (Office); CL165|Path (Cardboard Box Area); CL166|Path (Cart Area); CL167|Path (Issuing Area) **Cross Aisle Path:** CL168|1–2; CL169|2–3; CL170|3–4; CL171|4–5 **Aisle Path:** CL172|1; CL173|2; CL174|3; CL175|4; CL176|5; CL177|Front; CL178|Back **Other:** CL179|Another Location; CL180|Location Unknown
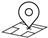	CC12	Location–Cart		X	27	**Main Area:** CL181|Transition between Areas; CL182|Office; CL183|Cart Area; CL184|Cardboard Box Area; CL185|Base; CL186|Packing/Sorting Area; CL187|Issuing/Receiving Area; CL188|Path; CL189|Cross Aisle Path; CL190|Aisle Path **Path:** CL191|Path (Office); CL192|Path (Cardboard Box Area); CL193|Path (Cart Area); CL194|Path (Issuing Area) **Cross Aisle Path:** CL195|1–2; CL196|2–3; CL197|3–4; CL198|4–5 **Aisle Path:** CL199|1; CL200|2; CL201|3; CL202|4; CL203|5; CL204|Front; CL205|Back **Other:** CL206|Another Location; CL207|Location Unknown

**Table 7:** Effort for annotating and revising 3, 444, 327 frames of video footage (31:55:26 hh:mm:ss) from S01–S18 for every CC. The ratio indicates the average time required to annotate or revise one minute of video footage, calculated as the total annotation or revision time divided by 31:55:26 hh:mm:ss.

Class Category		Annotation		Revision	
		Total	Ratio	Total	Ratio
		[hh:mm:ss]			
CC01	Main Activity	172:35:30	0:05:24	–	–
CC02	Sub-Activity–Legs	278:44:27	0:08:44	68:12:31	0:02:08
CC03	Sub-Activity–Torso	108:12:37	0:03:23	76:13:01	0:02:23
CC04	Sub-Activity–Left Hand	384:24:34	0:12:02	71:15:00	0:02:14
CC05	Sub-Activity–Right Hand	378:12:41	0:11:51	73:46:51	0:02:19
CC06	Order	3:13:20	0:00:06	1:01:40	0:00:02
CC07	Information Technology				
CC08	High-Level Process				
CC09	Mid-Level Process	129:15:32	0:04:03	39:20:41	0:01:14
CC10	Low-Level Process				
CC11	Location–Human	92:08:38	0:02:53	22:51:42	0:00:43
CC12	Location–Cart	25:19:00	0:00:48	8:30:55	0:00:16
**Total**	**All Categories**	**1572:06:19**	**0:49:15**	**361:12:21**	**0:11:19**

**Table 8:** Strength of agreement over annotations and revisions divided by class categories. As the labels of classes CC04 and CC05 are semantically equivalent, test annotation was performed exclusively for *Sub-Activity–Left Hand* (CC04). It is evident that the label definitions are equivalent; therefore, the resulting Light’s kappa value can be directly applied to the *Sub-Activity–Right Hand* (CC05).

Class Category		Cohen’s/Light’s Kappa [%]	
ID	Name	Annotation	Revision
CC01	Main Activity	75.77	80.59
CC02	Sub-Activity–Legs	60.99	78.27
CC03	Sub-Activity–Torso	40.83	81.61
CC04	Sub-Activity–Left Hand	71.32	78.35
CC05	Sub-Activity–Right Hand		
CC06	Order	95.44	99.88
CC07	Information Technology	95.20	99.86
CC08	High-Level Process	94.53	99.85
CC09	Mid-Level Process	89.63	98.63
CC10	Low-Level Process	73.25	90.95
CC11	Location–Human	88.54	98.04
CC12	Location–Cart	92.47	98.16

**Table 9:** Recall [%] and precision [%] of human activity recognition (HAR) with predicting the activity classes using Softmax on the DaRA IMU dataset.

Main Activity	Metric	
	Recall	Precision
Confirm with Pen	91.18	3.05
Confirm with Screen	0.00	0.00
Confirm with Button	57.50	4.01
Scan	18.97	7.42
Pull	78.16	66.89
Push	74.21	90.38
Handling Upwards	54.17	61.39
Handling Centered	71.99	84.23
Handling Downwards	66.45	54.37
Walking	80.00	75.36
Standing	81.88	67.83

**Table 10:** The overall accuracy [%] and wF1 [%] of HAR using Softmax for predicting activities k and Sigmoid for predicting an attribute vector a on the IMU of the DaRA dataset. An attribute representation a is a combination of sub-activity labels with a∈B, creating a sort of semantic description of an activity.

Metric	Softmax	Attributes
Acc [%]	72.12	74.62
wF1 [%]	70.40	73.70

**Table 11:** Confusion matrix from the class predictions using tCNN-IMU with the softmax layer.

Main Activity	Confusion Matrix										
	Confirm with			Scan	Pull	Push	Handling			Walk.	Stand.
	Pen	Screen	Button				Up.	Cen.	Down.		
**Confirm with Pen**	124	0	0	32	7	43	1948	1623	83	121	87
**Confirm with Screen**	0	0	5	0	1	12	45	367	42	106	107
**Confirm with Button**	0	0	46	3	0	3	161	609	167	155	2
**Scan**	0	63	1	291	6	9	340	1924	534	156	600
**Pull**	0	0	0	0	2347	775	6	374	3	3	1
**Push**	0	0	0	0	249	6276	0	411	0	3	5
**Handling Upwards**	11	1	12	191	19	58	7899	4513	14	119	29
**Handling Centered**	1	26	4	502	319	883	3251	64,222	2079	3157	1805
**Handling Down.**	0	1	1	51	0	2	63	4858	6711	467	190
**Walking**	0	0	6	2	15	303	155	5398	62	20,004	598
**Standing**	0	0	5	462	40	93	714	4906	404	714	15,473

**Figure A2:**
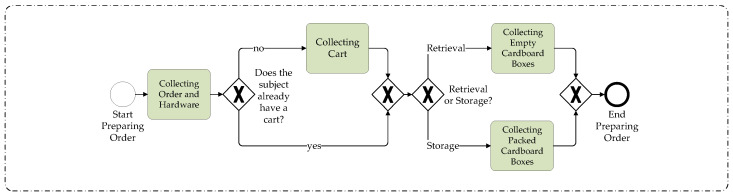
Idealized BPMN of the mid-level process *Preparing Order* with its low-level processes.

**Figure A3:**
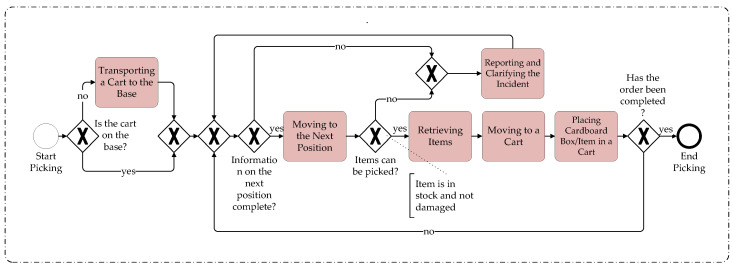
Idealized BPMN of the mid-level processes *Picking–Travel Time* and *Picking–Pick Time* with its low-level processes.

**Figure A4:**
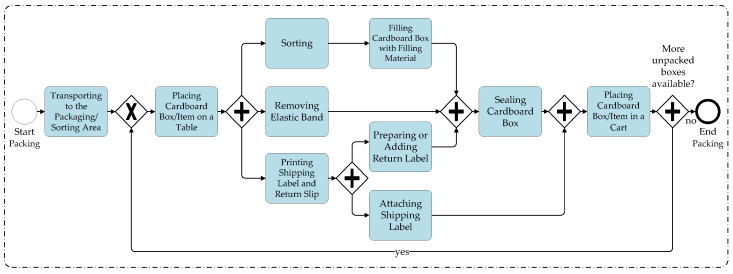
Idealized BPMN of the mid-level process *Packing* with its low-level processes.

**Figure A5:**
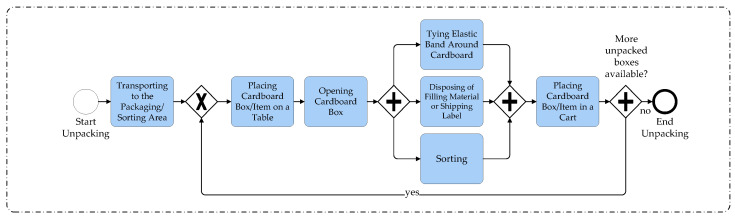
Idealized BPMN of the mid-level process *Unpacking* with its low-level processes.

**Figure A6:**
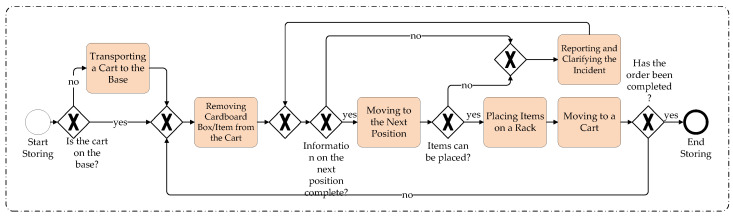
Idealized BPMN of the mid-level processes *Storing–Travel Time* and *Storing–Store Time* with its low-level processes.

**Figure A7:**
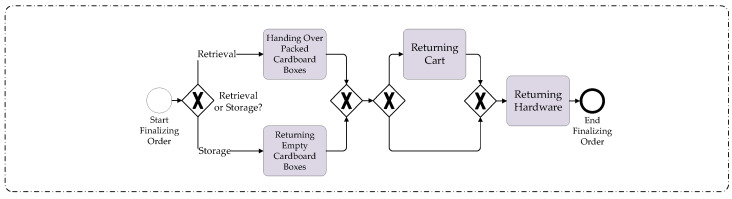
Idealized BPMN of the mid-level process *Finalizing Order* with its low-level processes.

## Data Availability

The dataset described and used in this work is freely available on Zenodo: [15].

## Abbreviations

The following abbreviations are used in this manuscript:

ADL	Activities of Daily Living
BLE	Bluetooth Low Energy
BPMN	Business Process Model and Notation
CAARL	Context-Aware Activity Recognition in Logistics
CC	Class Category
CL	Class Label
CNN	Convolutional Neural Network
DaRA	Data Fusion for advanced Research in industrial Applications
FN	False Negative
FP	False Positive
fps	Frame per Second
FPV	First-Person View
HAR	Human Activity Recognition
HCR	Human Context Recognition
HMM	Hidden Markov Model
Hz	Herz
ID	Identification
IMU	Inertial Measurement Unit
IT	Information Technology
LARa	Logistic Activity Recognition Challenge
LSTM	Long Short-Term Memory
MoCap	Motion Capture
Nr.	Number
P	Precision
PDT	Portable Data Terminal
PH	Person-Hours
R	Recall
RGB	Red–Green–Blue (refering to colored video)
RGB-D	Red–Green–Blue and Depth (refering to colored video with depth information)
RSSI	Received Signal Strength Indicator
TN	True Negative
tcnn	Temporal Convolutional Neural Network
TP	True Positive
TPV	Third-Person View
WMS	Warehouse Management System
